# Supplementary material for: New Perspectives of Multiplex Mass Spectrometry Blood Protein Quantification on Microsamples in Biological Monitoring of Elderly Patients
Source: Int J Mol Sci. 2023 Apr 10;24(8):6989. doi: 10.3390/ijms24086989 (PMC10139225; doi:10.3390/ijms24086989)
Supplement: Supplementary file 1 [file ijms-24-06989-s001.zip › ijms-2282054-supplementary.pdf]

Supplementary information

Supplementary information 1
Table S1: List of proteins, corresponding peptides 2
Table S2: List of the quantifications of the 62 proteins according to the different types of samples (plasma micro samples and Dried Blood Spots DBS) for each patient and expressed in z-score (cf Excelfile Supplemental Table 2). 4
Table S3: List of proteins and analytical performance of microsample multiplex analyses 5
Table S4: Descriptive and statistic results when comparing low IADL group to high IADL group. 7
Table S5: Optimized collision energy for the different peptide ions. 13
Figure S1: LC-MRM chromatogram and calibration curves 32
Sup Methods 32
Chemicals used 32
Preparation of samples before mass spectrometry analysis 33
Mass Spectrometry Analysis 33
Stable isotopically labelled peptide mix 34
Precision study 38

Table S1: List of proteins, corresponding peptides

Protein names, sequence of the proteotypic peptides used for its mass spectrometry detection and Uniprot access number are reported. Link the the LabTestOnline description of the protein are provided, as well as the reference number in the French nomenclature of biological test and the corresponding reimbursement cost indicate in B (which corresponds to the cost unit (0.27€ in 2021) of the biological assays in France). (*) the five proteins corresponding the clinical trial main outcome.

Protein Name	Peptide	UniProt KB Accession No.	Lab-test-online®	Code (NABM)	Cost (B)
Afamin	DADPDTFFAK	P43652		/	/
Albumin *	LVNEVTEFAK	P02768	https://labtestsonline.org/tests/albumin	1806	6
Alpha-1-acid glycoprotein 1 (AAG) *	TEDTIFLR	P02763		1808	30
Alpha-1-antichymotrypsin	NLAVSQVVHK	P01011		1807	30
Alpha-1B-glycoprotein	GVTFLLR	P04217	https://labtestsonline.org/tests/protein-electrophoresis-immunofixation-electrophoresis	/	/
Alpha-2-antiplasmin	LGNQEPGGQTALK	P08697		E054	140
Alpha-2-HS-glycoprotein	FSVVYAK	P02765		/	/
Alpha-2-macroglobulin	AIGYLNTGYQR	P01023	https://labtestsonline.org/tests/protein-electrophoresis-immunofixation-electrophoresis	1805	25
Antithrombin-III	DDLYVSDAFHK	P01008	https://labtestsonline.org/tests/antithrombin	0188	38
Apolipoprotein A-I	ATEHLSTLSEK	P02647	https://labtestsonline.org/tests/apo-i	1603	7
Apolipoprotein A-IV	LLPHANEVSQK	P06727		/	/
Apolipoprotein B-100	TGISPLALIK	P04114	https://labtestsonline.org/tests/apo-b	1602	7
Apolipoprotein C-II	TAAQNLYEK	P02655		L171	40
Apolipoprotein D	VLNQELR	P05090		/	/
Apolipoprotein E	LGPLVEQGR	P02649	https://labtestsonline.org/tests/apoe-genotyping-cardiovascular-disease	L034	200
Apolipoprotein M	AFLLTPR	O95445		/	/
Apolipoprotein(a)	GTYSTTVTGR	P08519	https://labtestsonline.org/tests/lipoprotein-a	/	/
Beta-2-glycoprotein 1	ATVVYQGER	P02749		/	/
Beta-2-microglobulin	IQVYSR	P61769	https://labtestsonline.org/tests/beta-2-microglobulin-kidney-disease	324	/
Beta-Ala-His dipeptidase	SVVLIPLGAVDDGEHSQNEK	Q96KN2		/	/
C4b-binding protein alpha chain	EDVYVVGTVLR	P04003		/	/
CD5 antigen-like	LVGGLHR	O43866		/	/
Cholinesterase	YLTLNTESTR	P06276	https://labtestsonline.org/tests/cholinesterase-tests	/	/

Clusterin	ELDESLQVAER	P10909		/	/
Coagulation factor X	TGIVSGFGR	P00742	https://labtestsonline.org/tests/coagulation-factors	0187	40
Coagulation factor XII	TEQAAVAR	P00748	https://labtestsonline.org/tests/coagulation-factors	/	/
Complement C1q subcomponent subunit B	IAFSATR	P02746	https://labtestsonline.org/tests/complement	K194	70
Complement C1q subcomponent subunit C	FQSVFTVTR	P02747	https://labtestsonline.org/tests/complement	K194	70
Complement C1r subcomponent	GLTLHLK	P00736		K196	100
Complement C2	HAFILQDTK	P06681	https://labtestsonline.org/tests/complement	K196	100
Complement C3	SGIPIVTSPYQIHFTK	P01024	https://labtestsonline.org/tests/complement	1811	25
Complement C4-B	GSFEFPVGDAVSK	P0C0L5	https://labtestsonline.org/tests/complement	K194	70
Complement C5	VFQFLEK	P01031		K194	70
Complement component C8 beta chain	SDLEVAHYK	P07358		K194	70
Complement component C9	LSPIYNLVPVK	P02748		K194	70
Complement factor D	THHDGAITER	P00746		/	/
Complement factor I	HGNTDSEGIVEVK	P05156		K194	70
Corticosteroid-binding globulin	ITQDAQLK	P08185		/	/
C-reactive protein *	ESDTSYVSLK	P02741	https://labtestsonline.org/tests/c-reactive-protein-crp	1804	8
Fibrinogen alpha chain	ESSSHHPGIAEFPSR	P02671	https://labtestsonline.org/tests/fibrinogen	0174	15
Fibulin-1	GYHLNEEGTR	P23142		/	/
Gelsolin	AGALNSNDAFVLK	P06396		/	/
Haptoglobin	DYAEVGR	P00738	https://labtestsonline.org/tests/haptoglobin	1813	20
Hemoglobin subunit alpha	TYFPHFDLSHGSAQVK	P69905	https://labtestsonline.org/tests/hemoglobin	/	/
Hemopexin	NFPSPVDAAFR	P02790		/	/
Heparin cofactor 2	SVNDLYIQK	P05546	https://labtestsonline.org/tests/heparin-anti-xa	/	/
Insulin-like growth factor-binding protein 3	FLNVLSPR	P17936		/	/
Insulin-like growth factor-binding protein complex acid labile subunit	LAELPADALGPLQR	P35858		/	/
Inter-alpha-trypsin inhibitor heavy chain H2	FYNQVSTPLLR	P19823		/	/
Lipopolysaccharide-binding protein	ITLPDFTGDLR	P18428		/	/
Lumican	FNALQYLR	P51884		/	/
Neuropilin-2	ALQVVR	O60462		/	/
Pigment epithelium-derived factor	TVQAVLTVPK	P36955		/	/

Prealbumin *	AADDTWEPFASGK	P02766	https://labtestsonline.org/tests/prealbumin	1817	19
Protein AMBP	ETLLQDFR	P02760		/	/
Prothrombin	ELLESYIDGR	P00734		E086	140
Retinol-binding protein 4	YWGVASFLQK	P02753		1818	35
Serum amyloid A-4 protein	GNYDAAQR	P35542		K162	70
Thyroxine-binding globulin	AVLHIGEK	P05543		K109	150
Transferrin	DGAGDVAFVK	P02787	https://labtestsonline.org/tests/transferrin-and-iron-binding-capacity-tibc-uibc	1819	14
Vitamin D-binding protein	ELPEHTVK	P02774		/	/
Vitamin K-dependent protein S	VYFAGFPR	P07225		/	/

Table S2: List of the quantifications of the 62 proteins according to the different types of samples (plasma micro samples and Dried Blood Spots DBS) for each patient and expressed in z-score (cf Excelfile Supplemental Table S2).

Table S3: List of proteins and analytical performance of microsample multiplex analyses

Protein names, analytical values of the mass spectrometry including the lower limit of quantification (LOQ) as provided by the PeptiQuant™ Biomarker Assessment Kit (BAK-76) from CIL (Cambridge Isotope Laboratories, Tewksbury, Massachusetts) except the 5 proteins added on this assay. Intra and inter-assay coefficient of variations (CV in %) which is the ratio of the standard deviation to the mean were provided.

Protein Name	Lower LOQ (ng/mL)	Intra-assay CV for plasma microsample	Intra-assay CV for DBS	Inter-assay CV using plasma QC
Afamin	314	8.0	11.1	10.1
Albumin	266	17.7	9.2	13.5
Alpha-1-acid glycoprotein 1 (AAG)	33600	1.6	1.2	12.4
Alpha-1-antichymotrypsin	4633	2.2	2.8	1.8
Alpha-1B-glycoprotein	2085	2.4	2.0	1.3
Alpha-2-antiplasmin	300	2.4	4.4	2.0
Alpha-2-HS-glycoprotein	1314	1.6	1.5	2.3
Alpha-2-macroglobulin	61976	1.7	2.1	1.5
Antithrombin-III	2862	10.2	11.4	6.1
Apolipoprotein A-I	12365	2.8	2.2	1.6
Apolipoprotein A-IV	1842	4.7	3.3	13.6
Apolipoprotein B-100	88601	1.4	3.1	2.5
Apolipoprotein C-II	55	6.6	5.2	18.4
Apolipoprotein D	2167	2.7	1.6	2.1
Apolipoprotein E	1763	2.0	2.0	1.7
Apolipoprotein M	2259	2.6	3.4	2.3
Apolipoprotein(a)	1288	6.8	9.4	14.4
Beta-2-glycoprotein 1	584	3.0	1.7	6.7
Beta-2-microglobulin	145	8.3	13.8	22.4
Beta-Ala-His dipeptidase	406	6.0	4.4	NA
C4b-binding protein alpha chain	2100	9.0	4.5	7.0
CD5 antigen-like	406	3.4	5.4	7.1
Cholinesterase	809	9.9	12.9	6.6
Clusterin	67381	2.1	2.6	5.1
Coagulation factor X	95	3.6	3.5	3.8
Coagulation factor XII	1664	5.3	7.7	1.5
Complement C1q subcomponent subunit B	137	2.4	3.3	NA
Complement C1q subcomponent subunit C	4023	4.6	5.3	3.5
Complement C1r subcomponent	302	7.0	4.8	7.7
Complement C2	396	8.4	12.7	11.0
Complement C3	238216	2.1	2.7	2.9
Complement C4-B	5353	4.0	2.6	2.6
Complement C5	781	5.1	19.0	4.4
Complement component C8 beta chain	100	8.2	5.9	6.2
Complement component C9	1470	4.5	2.1	2.2
Complement factor D	306	4.1	7.4	3.0
Complement factor I	945	4.5	3.9	5.9
Corticosteroid-binding globulin	406	2.6	6.6	6.7

C-reactive protein	251	3.8	6.6	5.7
Fibrinogen alpha chain	26190	2.7	1.7	1.4
Fibulin-1	162	6.0	6.6	8.6
Gelsolin	2034	4.9	4.4	3.3
Haptoglobin	328	2.7	2.8	9.4
Hemoglobin subunit alpha	7370	20.3	10.9	6.0
Hemopexin	11896	1.9	1.4	2.2
Heparin cofactor 2	735	1.6	2.3	2.8
Insulin-like growth factor-binding protein 3	98	3.1	10.4	2.4
Insulin-like growth factor-binding protein complex acid labile subunit	2394	5.7	7.8	5.0
Inter-alpha-trypsin inhibitor heavy chain H2	7037	2.6	4.0	2.1
Lipopolysaccharide-binding protein	469	8.4	8.9	10.0
Lumican	742	2.4	3.0	3.6
Neuropilin-2	112	4.7	5.4	9.1
Pigment epithelium-derived factor	3107	2.8	4.3	3.1
Prealbumin	2752	3.2	2.8	10.3
Protein AMBP	1546	2.6	3.5	4.0
Prothrombin	18014	6.4	8.4	5.2
Retinol-binding protein 4	1476	3.5	4.0	3.9
Serum amyloid A-4 protein	135	2.7	2.8	5.0
Thyroxine-binding globulin	110	3.8	3.4	3.8
Transferrin	4833	1.6	1.6	1.9
Vitamin D-binding protein	3819	2.8	2.3	4.2
Vitamin K-dependent protein S	1266	2.9	6.6	2.6

Table S4: Descriptive and statistic results when comparing low IADL group to high IADL group. Abbreviations: Dried Blood Spots (DBS), Instrumental Activities of Daily Living (IADL)

Variable	IADL<5			IADL>=5			
	n	Median	Average Rank	n	Median	Average Rank	P [a]
**Apolipoprotein D DBS**	23	0.544	23.739	15	0.400	13	0.0029
**Apolipoprotein D Plasma**	23	5.375	23.435	15	3.603	13.467	0.0061
**Apolipoprotein E DBS**	23	0.538	23.087	15	0.442	14	0.0130
**Apolipoprotein E Plasma**	23	6.520	22.304	15	4.884	15.200	0.0552
**Alpha-2-macroglobulin DBS**	23	0.484	17.391	15	0.573	22.733	0.1534
**Alpha-2-macroglobulin Plasma**	23	6.407	15.652	15	7.802	25.400	0.0074
**Insulin growth factor-binding protein complex acide labile subunit DBS**	23	0.344	22.696	15	0.205	14.600	0.0280
**Insulin growth factor-binding protein complex acide labile subunit Plasma**	23	4.603	20.870	15	3.562	17.400	0.3590
**Apolipoprotein(a) DBS**	23	1.613	20.696	15	1.558	17.667	0.4247
**Apolipoprotein(a) Plasma**	23	6.338	20.696	15	4.859	17.667	0.4247
**Apolipoprotein M DBS**	23	1.151	21.870	15	1.010	15.867	0.1074
**Apolipoprotein M Plasma**	23	4.053	22	15	3.607	15.667	0.0889
**Beta-2-glycoprotein 1 DBS**	23	0.774	18.783	15	0.893	20.600	0.6368
**Beta-2-glycoprotein 1 Plasma**	23	11.305	19.130	15	10.568	20.067	0.8135
**Beta-2-microglobulin DBS**	23	0.469	19.652	15	0.467	19.267	0.9295
**Beta-2-microglobulin Plasma**	23	7.163	19.956	15	5.936	18.800	0.7680
**Beta-Ala-His_dipeptidase DBS**	23	0.934	18.739	15	0.976	20.667	0.6158
**Beta-Ala-His_dipeptidase Plasma**	23	5.634	17.261	15	8.022	22.933	0.1289

**C4b-binding_protein_alpha_chain DBS**	23	2.037	19.217	15	1.891	19.933	0.8596
**C4b-binding_protein_alpha_chain Plasma**	23	10.469	18.435	15	11.627	21.133	0.4782
**CD5_antigen-like DBS**	23	0.217	19.783	15	0.195	19.067	0.8596
**CD5_antigen-like Plasma**	23	3.106	19.043	15	3.460	20.200	0.7680
**Cholinesterase DBS**	23	0.396	17.043	15	0.677	23.267	0.0947
**Cholinesterase Plasma**	23	5.812	18.609	15	5.673	20.867	0.5548
**Clusterin DBS**	23	6.298	19.783	15	6.556	19.067	0.8596
**Clusterin Plasma**	23	36.568	18.826	15	36.981	20.533	0.6580
**Coagulation_factor_X DBS**	23	0.314	20.217	15	0.323	18.400	0.6368
**Coagulation_factor_X Plasma**	23	3.408	20.391	15	3.224	18.133	0.5548
**Coagulation_factor_XII DBS**	23	0.612	19.783	15	0.580	19.067	0.8596
**Coagulation_factor_XII Plasma**	23	3.846	19.609	15	3.829	19.333	0.9530
**Complement_C1q_subcomponent_subunit_B DBS**	23	0.385	18.348	15	0.499	21.267	0.4422
**Complement_C1q_subcomponent_subunit_B Plasma**	23	9.603	19.956	15	9.226	18.800	0.7680
**Complement_C1q_subcomponent_subunit_C DBS**	23	0.404	18.870	15	0.420	20.467	0.6796
**Complement_C1q_subcomponent_subunit_C Plasma**	23	12.897	19.783	15	12.970	19.067	0.8596
**Complement_C1r_subcomponent DBS**	23	0.931	20.174	15	0.868	18.467	0.6580
**Complement_C1r_subcomponent Plasma**	23	11.523	19.087	15	11.265	20.133	0.7907
**Complement_C2 DBS**	23	2.023	21.043	15	1.742	17.133	0.2999

**Complement_C2 Plasma**	23	10.219	20.435	15	9.892	18.067	0.5351
**Complement_C3 DBS**	23	0.541	19.826	15	0.547	19	0.8365
**Complement_C3 Plasma**	23	7.086	19.348	15	7.052	19.733	0.9295
**Complement_C4-B DBS**	23	0.926	21.609	15	0.789	16.267	0.1534
**Complement_C4-B Plasma**	23	12.071	21.696	15	10.492	16.133	0.1367
**Complement_C5 DBS**	23	0.663	19.522	15	0.697	19.467	1
**Complement_C5 Plasma**	23	6.914	19.522	15	6.873	19.467	1
**Complement_component_C8_beta_chain DBS**	23	0.887	19.826	15	0.824	19	0.8365
**Complement_component_C8_beta_chain Plasma**	23	8.654	18.304	15	9.708	21.333	0.4247
**Complement_component_C9 DBS**	23	0.741	19.043	15	0.751	20.200	0.7680
**Complement_component_C9 Plasma**	23	6.004	19.391	15	5.862	19.667	0.9530
**Complement_factor_D_DBS**	23	0.157	19.565	15	0.159	19.400	0.9765
**Complement_factor_D_Plasma**	23	1.162	19.174	15	1.202	20	0.8365
**Complement_factor_I _DBS**	23	0.162	21.348	15	0.136	16.667	0.2126
**Complement_factor_I_Plasma**	23	1.304	21.652	15	0.979	16.200	0.1449
**Corticosteroid-binding_globulin_DBS**	23	0.251	19.435	15	0.241	19.600	0.9765
**Corticosteroid-binding_globulin_Plasma**	23	4.052	20.870	15	3.828	17.400	0.359
**C Reactive Protein DBS**	23	0.056	22	15	0.028	15.667	0.0889
**C Reactive Protein Plasma**	23	0.614	21	15	0.355	17.200	0.314

**Fibrinogen_alpha_chain DBS**	23	1.342	18.652	15	1.376	20.800	0.5748
**Fibrinogen_alpha_chain Plasma**	23	13.097	18.087	15	15.266	21.667	0.3436
**Fibulin-1 DBS**	23	0.704	20.130	15	0.585	18.533	0.6796
**Fibulin-1 Plasma**	23	6.487	20.696	15	6.565	17.667	0.4247
**Gelsolin DBS**	23	1.179	19.391	15	1.168	19.667	0.953
**Gelsolin Plasma**	23	4.183	20.261	15	3.993	18.333	0.6158
**Haptoglobin DBS**	23	8.477	21.217	15	7.230	16.867	0.2475
**Haptoglobin Plasma**	23	90.798	20.739	15	62.836	17.600	0.4077
**Hemoglobin_subunit_alpha DBS**	23	1110.223	20.130	15	1101.770	18.533	0.6796
**Hemoglobin_subunit_alpha Plasma**	23	3.916	20	15	2.847	18.733	0.7456
**Heparin_cofactor_ DBS**	23	1.276	18.956	15	1.389	20.333	0.7233
**Heparin_cofactor_Plasma**	23	16.244	19.174	15	16.568	20	0.8365
**Insulin-like_growth_factor-binding_protein_3_ DBS**	23	0.470	20.087	15	0.452	18.600	0.7013
**Insulin-like_growth_factor-binding_protein_3_Plasma**	23	2.996	20.130	15	2.828	18.533	0.6796
**Inter-alpha-trypsin_inhibitor_heavy_chain_H2 DBS**	23	1.216	19.956	15	1.338	18.800	0.7680
**Inter-alpha-trypsin_inhibitor_heavy_chain_H2 Plasma**	23	8.130	20.739	15	8.104	17.600	0.4077

**Lipopolysaccharide-binding_protein_DBS**	23	2.199	19.956	15	2.123	18.800	0.7680
**Lipopolysaccharide-binding_protein_Plasma**	23	46.817	19.913	15	47.120	18.867	0.7907
**Lumican_DBS**	23	4.869	18.956	15	5.230	20.333	0.7233
**Lumican_Plasma**	23	3.344	18.130	15	3.440	21.600	0.3590
**Neuropilin-2_DBS**	23	0.271	20.435	15	0.251	18.067	0.5351
**Neuropilin-2_Plasma**	23	3.520	20.522	15	3.281	17.933	0.4968
**Alpha-1-acid glycoprotein 1 DBS**	23	0.351	19.913	15	0.348	18.867	0.7907
**Alpha-1-acid glycoprotein 1 Plasma**	23	6.802	21.826	15	6.355	15.933	0.1143
**Pigment epithelium-derived factor DBS**	23	0.038	20.652	15	0.033	17.733	0.4422
**Pigment epithelium-derived factor Plasma**	23	0.211	20.261	15	0.201	18.333	0.6158
**Protein_AMBP DBS**	23	0.789	20.261	15	0.636	18.333	0.6158
**Protein_AMBP Plasma**	23	5.439	19.739	15	5.242	19.133	0.8828
**Prothrombin DBS**	23	0.421	19.826	15	0.451	19	0.8365
**Prothrombin Plasma**	23	3.805	21.130	15	3.509	17	0.2729
**Transferrin DBS**	23	2.330	20.478	15	2.271	18	0.5158
**Transferrin Plasma**	23	26.980	20.087	15	22.501	18.600	0.7013
**Serum_amyloid_A-4_protein DBS**	23	2.476	21.913	15	1.903	15.800	0.1009
**Serum_amyloid_A-4_protein Plasma**	23	11.938	21.348	15	8.433	16.667	0.2126
**Retinol-binding protein 4 DBS**	23	1.570	19.217	15	1.938	19.933	0.8596
**Retinol-binding protein 4 Plasma**	23	54.673	19.478	15	48.323	19.533	1
**Hemopexin DBS**	23	2.810	19.348	15	2.552	19.733	0.9295

**Hemopexin Plasma**	23	28.835	18.826	15	29.084	20.533	0.6580
**Thyroxine-binding_globulin DBS**	23	0.462	19.043	15	0.454	20.200	0.7680
**Thyroxine-binding_globulin Plasma**	23	4.014	17.783	15	4.113	22.133	0.2475
**Transthyretin DBS**	23	3.655	19.652	15	3.235	19.267	0.9295
**Transthyretin Plasma**	23	2.743	19.609	15	2.900	19.333	0.9530
**Vitamin_D-binding_protein DBS**	23	0.509	20.217	15	0.482	18.400	0.6368
**Vitamin_D-binding_protein Plasma**	23	3.549	19.826	15	3.570	19	0.8365
**Vitamin_K-dependent_protein_S DBS**	23	0.407	19.913	15	0.384	18.867	0.7907
**Vitamin_K-dependent_protein_S Plasma**	23	5.297	19.609	15	5.883	19.333	0.9530

[a] Mann-Whitney test

MedCalc® Statistical Software version 20.210 (MedCalc Software Ltd, Ostend, Belgium; https://www.medcalc.org; 2022)

Table S5: Optimized collision energy for the different peptide ions.

Protein name and corresponding peptide on endogenous form (.light) or isotopically labelled (.heavy). On each peptide transition parameters were indicated: precursor ion, fragment ion, collision energy used to generate this fragmentation and the indication of the fragment ion type. In addition, the retention time of the peptide was indicated.

Protein	Peptide	Precursor Ion (m/z)	Product Ion (m/z)	Collision Energy (V)	Ion Type	Retention Time (min)	Collision Energy	Ret Time (min)	Delta Ret Time	Ion Name
Afamin	DADPDTFFAK.light	563,76	302,10	-22	b3	20	-22	20	3	b3
Afamin	DADPDTFFAK.light	563,76	825,41	-22	y7	20	-22	20	3	y7
Afamin	DADPDTFFAK.light	563,76	413,21	-22	y7	20	-22	20	3	y7
Afamin	DADPDTFFAK.heavy	567,76	302,10	-22	b3	20	-22	20	3	b3
Afamin	DADPDTFFAK.heavy	567,76	833,43	-22	y7	20	-22	20	3	y7
Afamin	DADPDTFFAK.heavy	567,76	417,22	-22	y7	20	-22	20	3	y7
Alpha-1B-glycoprotein	GVTFLLR.light	403,25	649,40	-16	y5	25	-16	25	3	y5
Alpha-1B-glycoprotein	GVTFLLR.light	403,25	401,29	-16	y3	25	-16	25	3	y3
Alpha-1B-glycoprotein	GVTFLLR.light	403,25	325,21	-16	y5	25	-16	25	3	y5
Alpha-1B-glycoprotein	GVTFLLR.heavy	408,25	659,41	-16	y5	25	-16	25	3	y5
Alpha-1B-glycoprotein	GVTFLLR.heavy	408,25	411,30	-16	y3	25	-16	25	3	y3
Alpha-1B-glycoprotein	GVTFLLR.heavy	408,25	330,21	-16	y5	25	-16	25	3	y5
Alpha-1B-glycoprotein	LETPDFQLFK.light	619,33	894,47	-24	y7	33	-24	33	3	y7
Alpha-1B-glycoprotein	LETPDFQLFK.light	619,33	498,26	-24	y8	33	-24	33	3	y8
Alpha-1B-glycoprotein	LETPDFQLFK.light	619,33	447,74	-24	y7	33	-24	33	3	y7
Alpha-1B-glycoprotein	LETPDFQLFK.light	619,33	344,18	-24	b3	33	-24	33	3	b3
Alpha-1B-glycoprotein	LETPDFQLFK.heavy	623,33	902,49	-24	y7	33	-24	33	3	y7
Alpha-1B-glycoprotein	LETPDFQLFK.heavy	623,33	502,27	-24	y8	33	-24	33	3	y8
Alpha-1B-glycoprotein	LETPDFQLFK.heavy	623,33	451,75	-24	y7	33	-24	33	3	y7
Alpha-1B-glycoprotein	LETPDFQLFK.heavy	623,33	344,18	-24	b3	33	-24	33	3	b3
sp|P08697|A2AP_HUMAN	LGNQEPGGQTALK.light	656,85	771,44	-26	y8	9	-26	9	3	y8
sp|P08697|A2AP_HUMAN	LGNQEPGGQTALK.light	656,85	285,16	-26	b3	9	-26	9	3	b3
sp|P08697|A2AP_HUMAN	LGNQEPGGQTALK.light	656,85	413,21	-26	b4	9	-26	9	3	b4
sp|P08697|A2AP_HUMAN	LGNQEPGGQTALK.light	656,85	542,26	-26	b5	9	-26	9	3	b5
sp|P08697|A2AP_HUMAN	LGNQEPGGQTALK.heavy	660,85	779,45	-26	y8	9	-26	9	3	y8
sp|P08697|A2AP_HUMAN	LGNQEPGGQTALK.heavy	660,85	285,16	-26	b3	9	-26	9	3	b3
sp|P08697|A2AP_HUMAN	LGNQEPGGQTALK.heavy	660,85	413,21	-26	b4	9	-26	9	3	b4
sp|P08697|A2AP_HUMAN	LGNQEPGGQTALK.heavy	660,85	542,26	-26	b5	9	-26	9	3	b5
Alpha-2-HS-glycoprotein	FSVVYAK.light	407,23	235,11	-16	b2	16	-16	16	3	b2

Alpha-2-HS-glycoprotein	FSVVYAK.light	407,23	666,38	-16	y6	16	-16	16	3	y6
Alpha-2-HS-glycoprotein	FSVVYAK.light	407,23	579,35	-16	y5	16	-16	16	3	y5
Alpha-2-HS-glycoprotein	FSVVYAK.heavy	411,24	235,11	-16	b2	16	-16	16	3	b2
Alpha-2-HS-glycoprotein	FSVVYAK.heavy	411,24	674,40	-16	y6	16	-16	16	3	y6
Alpha-2-HS-glycoprotein	FSVVYAK.heavy	411,24	587,36	-16	y5	16	-16	16	3	y5
Alpha-2-macroglobulin	AIGYLNTGYQR.light	628,33	851,44	-25	y7	17	-25	17	3	y7
Alpha-2-macroglobulin	AIGYLNTGYQR.light	628,33	738,35	-25	y6	17	-25	17	3	y6
Alpha-2-macroglobulin	AIGYLNTGYQR.light	628,33	242,15	-25	b3	17	-25	17	3	b3
Alpha-2-macroglobulin	AIGYLNTGYQR.heavy	633,33	242,15	-25	b3	17	-25	17	3	b3
Alpha-2-macroglobulin	AIGYLNTGYQR.heavy	633,33	861,45	-25	y7	17	-25	17	3	y7
Alpha-2-macroglobulin	AIGYLNTGYQR.heavy	633,33	748,36	-25	y6	17	-25	17	3	y6
sp|P01008|ANT3_HUMAN	DDLYVSDAFHK.light	437,21	540,28	-15	y9	19	-15	19	3	y9
sp|P01008|ANT3_HUMAN	DDLYVSDAFHK.light	437,21	483,74	-15	y8	19	-15	19	3	y8
sp|P01008|ANT3_HUMAN	DDLYVSDAFHK.light	437,21	402,21	-15	y7	19	-15	19	3	y7
sp|P01008|ANT3_HUMAN	DDLYVSDAFHK.light	437,21	231,06	-15	b2	19	-15	19	3	b2
sp|P01008|ANT3_HUMAN	DDLYVSDAFHK.heavy	439,88	544,29	-15	y9	19	-15	19	3	y9
sp|P01008|ANT3_HUMAN	DDLYVSDAFHK.heavy	439,88	487,74	-15	y8	19	-15	19	3	y8
sp|P01008|ANT3_HUMAN	DDLYVSDAFHK.heavy	439,88	406,21	-15	y7	19	-15	19	3	y7
sp|P01008|ANT3_HUMAN	DDLYVSDAFHK.heavy	439,88	231,06	-15	b2	19	-15	19	3	b2
Apolipoprotein(a)	GTYSTTVTGR.light	521,76	721,38	-20	y7	7	-20	7	3	y7
Apolipoprotein(a)	GTYSTTVTGR.light	521,76	432,26	-20	y4	7	-20	7	3	y4
Apolipoprotein(a)	GTYSTTVTGR.light	521,76	442,73	-20	y8	7	-20	7	3	y8
Apolipoprotein(a)	GTYSTTVTGR.heavy	526,77	731,39	-20	y7	7	-20	7	3	y7
Apolipoprotein(a)	GTYSTTVTGR.heavy	526,77	442,26	-20	y4	7	-20	7	3	y4
Apolipoprotein(a)	GTYSTTVTGR.heavy	526,77	447,73	-20	y8	7	-20	7	3	y8
sp|P02647|APOA1_HUMAN	ATEHLSTLSEK.light	405,88	664,35	-14	y6	8	-14	8	3	y6
sp|P02647|APOA1_HUMAN	ATEHLSTLSEK.light	405,88	572,80	-14	y10	8	-14	8	3	y10
sp|P02647|APOA1_HUMAN	ATEHLSTLSEK.light	405,88	522,27	-14	y9	8	-14	8	3	y9
sp|P02647|APOA1_HUMAN	ATEHLSTLSEK.light	405,88	457,75	-14	y8	8	-14	8	3	y8
sp|P02647|APOA1_HUMAN	ATEHLSTLSEK.heavy	408,55	672,37	-14	y6	8	-14	8	3	y6
sp|P02647|APOA1_HUMAN	ATEHLSTLSEK.heavy	408,55	576,80	-14	y10	8	-14	8	3	y10
sp|P02647|APOA1_HUMAN	ATEHLSTLSEK.heavy	408,55	526,28	-14	y9	8	-14	8	3	y9

sp|P02647|APOA1_HUMAN	ATEHLSTLSEK.heavy	408,55	461,76	-14	y8	8	-14	8	3	y8
P06727_APOA4_HUMAN	LLPHANEVSQK.light	412,56	227,18	-14	b2	9	-14	9	3	b2
P06727_APOA4_HUMAN	LLPHANEVSQK.light	412,56	231,15	-14	b4	9	-14	9	3	b4
P06727_APOA4_HUMAN	LLPHANEVSQK.light	412,56	456,73	-14	y8	9	-14	9	3	y8
P06727_APOA4_HUMAN	LLPHANEVSQK.heavy	415,23	460,74	-14	y8	9	-14	9	3	y8
P06727_APOA4_HUMAN	LLPHANEVSQK.heavy	415,23	235,15	-14	y4	9	-14	9	3	y4
P06727_APOA4_HUMAN	LLPHANEVSQK.heavy	415,23	227,18	-14	b2	9	-14	9	3	b2
ApoB100	TGISPLALIK.light	506,82	272,16	-20	b3	32	-20	32	3	b3
ApoB100	TGISPLALIK.light	506,82	741,49	-20	y7	32	-20	32	3	y7
ApoB100	TGISPLALIK.light	506,82	654,45	-20	y6	32	-20	32	3	y6
ApoB100	TGISPLALIK.heavy	510,83	272,16	-20	b3	32	-20	32	3	b3
ApoB100	TGISPLALIK.heavy	510,83	749,50	-20	y7	32	-20	32	3	y7
ApoB100	TGISPLALIK.heavy	510,83	662,47	-20	y6	32	-20	32	3	y6
ApoC2	TAAQNLYEK.light	519,27	173,09	-20	b2	8	-20	8	3	b2
ApoC2	TAAQNLYEK.light	519,27	865,44	-20	y7	8	-20	8	3	y7
ApoC2	TAAQNLYEK.light	519,27	666,35	-20	y5	8	-20	8	3	y5
ApoC2	TAAQNLYEK.heavy	523,27	173,09	-20	b2	8	-20	8	3	b2
ApoC2	TAAQNLYEK.heavy	523,27	873,46	-20	y7	8	-20	8	3	y7
ApoC2	TAAQNLYEK.heavy	523,27	674,36	-20	y5	8	-20	8	3	y5
ApoE	LGPLVEQGR.light	484,78	588,31	-19	y5	14	-19	14	3	y5
ApoE	LGPLVEQGR.light	484,78	489,24	-19	y4	14	-19	14	3	y4
ApoE	LGPLVEQGR.light	484,78	399,73	-18,9	y7	14,21	-18,9	14,21	3	y7
ApoE	LGPLVEQGR.heavy	489,78	598,32	-18,9	y5	14,21	-18,9	14,21	3	y5
ApoE	LGPLVEQGR.heavy	489,78	499,25	-18,9	y4	14,21	-18,9	14,21	3	y4
ApoE	LGPLVEQGR.heavy	489,78	404,73	-18,9	y7	14,21	-18,9	14,21	3	y7
ApoE	AATVGSLAGQPLQER.light	499,94	244,13	-17,7	b3	18,31	-17,7	18,31	3	b3
ApoE	AATVGSLAGQPLQER.light	499,94	642,36	-17,7	y5	18,31	-17,7	18,31	3	y5
ApoE	AATVGSLAGQPLQER.light	499,94	578,31	-17,7	y11	18,31	-17,7	18,31	3	y11
ApoE	AATVGSLAGQPLQER.heavy	503,27	244,13	-17,7	b3	18,31	-17,7	18,31	3	b3
ApoE	AATVGSLAGQPLQER.heavy	503,27	652,37	-17,7	y5	18,31	-17,7	18,31	3	y5
ApoE	AATVGSLAGQPLQER.heavy	503,27	583,31	-17,7	y11	18,32	-17,7	18,32	3	y11
ApoM	AFLLTPR.light	409,25	599,39	-15,9	y5	21,44	-15,9	21,44	3	y5

ApoM	AFLLTPR.light	409,25	486,30	-15,9	y4	21,44	-15,9	21,44	3	y4
ApoM	AFLLTPR.light	409,25	373,22	-15,9	y3	21,44	-15,9	21,44	3	y3
ApoM	AFLLTPR.heavy	414,25	609,40	-15,9	y5	21,44	-15,9	21,44	3	y5
ApoM	AFLLTPR.heavy	414,25	496,31	-15,9	y4	21,42	-15,9	21,42	3	y4
ApoM	AFLLTPR.heavy	414,25	383,23	-15,9	y3	21,44	-15,9	21,44	3	y3
ApoD	VLNQELR.light	436,25	772,43	-16,9	y6	9,51	-16,9	9,51	3	y6
ApoD	VLNQELR.light	436,25	659,35	-16,9	y5	9,51	-16,9	9,51	3	y5
ApoD	VLNQELR.light	436,25	545,30	-16,9	y4	9,51	-16,9	9,51	3	y4
ApoD	VLNQELR.heavy	441,26	782,44	-16,9	y6	9,51	-16,9	9,51	3	y6
ApoD	VLNQELR.heavy	441,26	669,36	-16,9	y5	9,51	-16,9	9,51	3	y5
ApoD	VLNQELR.heavy	441,26	555,31	-16,9	y4	9,51	-16,9	9,51	3	y4
Beta-2-microglobulin	IQVYSR.light	383,22	652,34	-14,8	y5	8,54	-14,8	8,54	3	y5
Beta-2-microglobulin	IQVYSR.light	383,22	524,28	-14,8	y4	8,54	-14,8	8,54	3	y4
Beta-2-microglobulin	IQVYSR.light	383,22	425,21	-14,8	y3	8,7	-14,8	8,7	3	y3
Beta-2-microglobulin	IQVYSR.heavy	388,22	662,35	-14,8	y5	8,67	-14,8	8,67	3	y5
Beta-2-microglobulin	IQVYSR.heavy	388,22	534,29	-14,8	y4	8,65	-14,8	8,65	3	y4
Beta-2-microglobulin	IQVYSR.heavy	388,22	435,22	-14,8	y3	8,67	-14,8	8,67	3	y3
Beta-2-microglobulin	VNHVTLSQPK.light	374,88	351,18	-13	b3	7,95	-13	7,95	3	b3
Beta-2-microglobulin	VNHVTLSQPK.light	374,88	459,26	-13	y4	7,95	-13	7,95	3	y4
Beta-2-microglobulin	VNHVTLSQPK.light	374,88	512,28	-13	y9	7,95	-13	7,95	3	y9
Beta-2-microglobulin	VNHVTLSQPK.heavy	377,55	351,18	-13	b3	7,95	-13	7,95	3	b3
Beta-2-microglobulin	VNHVTLSQPK.heavy	377,55	467,27	-13	y4	7,95	-13	7,95	3	y4
Beta-2-microglobulin	VNHVTLSQPK.heavy	377,55	516,29	-13	y9	7,95	-13	7,95	3	y9
Beta-Ala-His_dipeptidase	SVVLIPLGAVDDGEHSQNEK.light	703,03	399,26	-25,2	b4	28,89	-25,2	28,89	3	b4
Beta-Ala-His_dipeptidase	SVVLIPLGAVDDGEHSQNEK.light	703,03	512,34	-25,2	b5	28,91	-25,2	28,91	3	b5
Beta-Ala-His_dipeptidase	SVVLIPLGAVDDGEHSQNEK.light	703,03	798,37	-25,2	y15	28,89	-25,2	28,89	3	y15
Beta-Ala-His_dipeptidase	SVVLIPLGAVDDGEHSQNEK.heavy	705,70	399,26	-25,2	b4	28,88	-25,2	28,88	3	b4
Beta-Ala-His_dipeptidase	SVVLIPLGAVDDGEHSQNEK.heavy	705,70	512,34	-25,2	b5	28,79	-25,2	28,79	3	b5
Beta-Ala-His_dipeptidase	SVVLIPLGAVDDGEHSQNEK.heavy	705,70	802,38	-25,2	y15	28,89	-25,2	28,89	3	y15
C4b-binding_protein_alpha_chain	EDVYVVGTVLR.light	625,34	344,15	-24,5	b3	25,56	-24,5	25,56	3	b3
C4b-binding_protein_alpha_chain	EDVYVVGTVLR.light	625,34	644,41	-24,5	y6	25,56	-24,5	25,56	3	y6
C4b-binding_protein_alpha_chain	EDVYVVGTVLR.light	625,34	545,34	-24,5	y5	25,56	-24,5	25,56	3	y5

C4b-binding_protein_alpha_chain	EDVYVVGTVLR.heavy	630,35	344,15	-24,5	b3	25,61	-24,5	25,61	3	b3
C4b-binding_protein_alpha_chain	EDVYVVGTVLR.heavy	630,35	654,42	-24,5	y6	25,56	-24,5	25,56	3	y6
C4b-binding_protein_alpha_chain	EDVYVVGTVLR.heavy	630,35	555,35	-24,5	y5	25,57	-24,5	25,57	3	y5
C4b-binding_protein_alpha_chain	LNNGEITQHR.light	394,54	541,28	-13,8	y4	5,14	-13,8	5,14	3	y4
C4b-binding_protein_alpha_chain	LNNGEITQHR.light	394,54	534,76	-13,8	y9	5,14	-13,8	5,14	3	y9
C4b-binding_protein_alpha_chain	LNNGEITQHR.light	394,54	477,74	-13,8	y8	5,14	-13,8	5,14	3	y8
C4b-binding_protein_alpha_chain	LNNGEITQHR.heavy	397,87	551,29	-13,8	y4	5,14	-13,8	5,14	3	y4
C4b-binding_protein_alpha_chain	LNNGEITQHR.heavy	397,87	539,77	-13,8	y9	5,14	-13,8	5,14	3	y9
C4b-binding_protein_alpha_chain	LNNGEITQHR.heavy	397,87	482,75	-13,8	y8	5,14	-13,8	5,14	3	y8
CD5_antigen-like	LVGGLHR.light	376,23	213,16	-14,5	b2	7,06	-14,5	7,06	3	b2
CD5_antigen-like	LVGGLHR.light	376,23	638,37	-14,5	y6	7,06	-14,5	7,06	3	y6
CD5_antigen-like	LVGGLHR.light	376,23	539,30	-14,5	y5	7,06	-14,5	7,06	3	y5
CD5_antigen-like	LVGGLHR.heavy	381,24	213,16	-14,5	b2	7,06	-14,5	7,06	3	b2
CD5_antigen-like	LVGGLHR.heavy	381,24	648,38	-14,5	y6	7,06	-14,5	7,06	3	y6
CD5_antigen-like	LVGGLHR.heavy	381,24	549,31	-14,5	y5	7,06	-14,5	7,06	3	y5
CRP	AFVFPK.light	354,71	637,37	-13,7	y5	19,59	-13,7	19,59	3	y5
CRP	AFVFPK.light	354,71	490,30	-13,7	y4	19,62	-13,7	19,62	3	y4
CRP	AFVFPK.light	354,71	391,23	-13,7	y3	19,62	-13,7	19,62	3	y3
CRP	AFVFPK.heavy	358,71	645,39	-13,7	y5	19,6	-13,7	19,6	3	y5
CRP	AFVFPK.heavy	358,71	498,32	-13,7	y4	19,6	-13,7	19,6	3	y4
CRP	AFVFPK.heavy	358,71	399,25	-13,7	y3	19,62	-13,7	19,62	3	y3
CRP	ESDTSYVSLK.light	564,77	696,39	-22,1	y6	13,89	-22,1	13,89	3	y6
CRP	ESDTSYVSLK.light	564,77	609,36	-22,1	y5	13,87	-22,1	13,87	3	y5
CRP	ESDTSYVSLK.light	564,77	446,30	-22,1	y4	13,88	-22,1	13,88	3	y4
CRP	ESDTSYVSLK.light	564,77	347,23	-22,1	y3	13,88	-22,1	13,88	3	y3
CRP	ESDTSYVSLK.heavy	567,78	702,41	-22,1	y6	13,91	-22,1	13,91	3	y6
CRP	ESDTSYVSLK.heavy	567,78	615,38	-22,1	y5	13,91	-22,1	13,91	3	y5
CRP	ESDTSYVSLK.heavy	567,78	452,32	-22,1	y4	13,89	-22,1	13,89	3	y4
CRP	ESDTSYVSLK.heavy	567,78	353,25	-22,1	y3	13,91	-22,1	13,91	3	y3
AAG	TEDTIFLR.light	497,76	764,43	-19,4	y6	20,18	-19,4	20,18	3	y6
AAG	TEDTIFLR.light	497,76	548,36	-19,4	y4	20,18	-19,4	20,18	3	y4
AAG	TEDTIFLR.light	497,76	435,27	-19,4	y3	20,18	-19,4	20,18	3	y3

AAG	TEDTIFLR.heavy	500,77	770,45	-19,4	y6	20,17	-19,4	20,17	3	y6
AAG	TEDTIFLR.heavy	500,77	554,38	-19,4	y4	20,18	-19,4	20,18	3	y4
AAG	TEDTIFLR.heavy	500,77	441,29	-19,4	y3	20,18	-19,4	20,18	3	y3
AAG	WFYIASAFR.light	580,80	664,38	-18	y6	35,76	-18	35,76	3	y6
AAG	WFYIASAFR.light	580,80	551,29	-18	y5	35,76	-18	35,76	3	y5
AAG	WFYIASAFR.light	580,80	480,26	-18	y4	35,76	-18	35,76	3	y4
AAG	WFYIASAFR.light	580,80	305,65	-18	b4	35,75	-18	35,75	3	b4
AAG	WFYIASAFR.heavy	585,80	674,39	-18	y6	35,75	-18	35,75	3	y6
AAG	WFYIASAFR.heavy	585,80	561,30	-18	y5	35,75	-18	35,75	3	y5
AAG	WFYIASAFR.heavy	585,80	490,26	-18	y4	35,75	-18	35,75	3	y4
AAG	WFYIASAFR.heavy	585,80	305,65	-18	b4	35,75	-18	35,75	3	b4
Prealbumin	AADDTWEPFASGK.light	697,81	606,32	-27,4	y6	21,64	-27,4	21,64	3	y6
Prealbumin	AADDTWEPFASGK.light	697,81	291,17	-27,4	y3	21,62	-27,4	21,62	3	y3
Prealbumin	AADDTWEPFASGK.light	697,81	474,18	-27,4	b5	21,62	-27,4	21,62	3	b5
Prealbumin	AADDTWEPFASGK.heavy	701,82	614,34	-27,4	y6	21,62	-27,4	21,62	3	y6
Prealbumin	AADDTWEPFASGK.heavy	701,82	299,18	-27,4	y3	21,62	-27,4	21,62	3	y3
Prealbumin	AADDTWEPFASGK.heavy	701,82	474,18	-27,4	b5	21,6	-27,4	21,6	3	b5
Prealbumin	ALGISPFHEHAEVVFTANDSGPR.light	613,56	645,30	-21,9	y6	25,59	-21,9	25,59	3	y6
Prealbumin	ALGISPFHEHAEVVFTANDSGPR.light	613,56	531,25	-21,9	y5	25,58	-21,9	25,58	3	y5
Prealbumin	ALGISPFHEHAEVVFTANDSGPR.light	613,56	323,15	-21,9	y6	25,6	-21,9	25,6	3	y6
Prealbumin	ALGISPFHEHAEVVFTANDSGPR.heavy	615,06	645,30	-21,9	y6	25,6	-21,9	25,6	3	y6
Prealbumin	ALGISPFHEHAEVVFTANDSGPR.heavy	615,06	531,25	-21,9	y5	25,59	-21,9	25,59	3	y5
Prealbumin	ALGISPFHEHAEVVFTANDSGPR.heavy	615,06	323,15	-21,9	y6	25,6	-21,9	25,6	3	y6
Albumin	LVNEVTEFAK.light	575,31	694,38	-22,5	y6	19,49	-22,5	19,49	3	y6
Albumin	LVNEVTEFAK.light	575,31	595,31	-22,5	y5	19,42	-22,5	19,42	3	y5
Albumin	LVNEVTEFAK.light	575,31	469,23	-22,5	y8	19,46	-22,5	19,46	3	y8
Albumin	LVNEVTEFAK.heavy	578,32	694,38	-22,5	y6	19,29	-22,5	19,29	3	y6
Albumin	LVNEVTEFAK.heavy	578,32	595,31	-22,5	y5	19,3	-22,5	19,3	3	y5
Albumin	LVNEVTEFAK.heavy	578,32	469,23	-22,5	y8	19,44	-22,5	19,44	3	y8
Cholinesterase	YLTLNTESTR.light	599,31	277,15	-23,5	b2	15,46	-23,5	15,46	3	b2
Cholinesterase	YLTLNTESTR.light	599,31	921,46	-23,5	y8	15,46	-23,5	15,46	3	y8
Cholinesterase	YLTLNTESTR.light	599,31	707,33	-23,5	y6	15,46	-23,5	15,46	3	y6

Cholinesterase	YLTLNTESTR.heavy	604,31	277,15	-23,5	b2	15,44	-23,5	15,44	3	b2
Cholinesterase	YLTLNTESTR.heavy	604,31	931,47	-23,5	y8	15,46	-23,5	15,46	3	y8
Cholinesterase	YLTLNTESTR.heavy	604,31	717,34	-23,5	y6	15,44	-23,5	15,44	3	y6
Clusterin	ELDESLQVAER.light	644,82	802,44	-25,3	y7	16,71	-25,3	16,71	3	y7
Clusterin	ELDESLQVAER.light	644,82	602,33	-25,3	y5	16,71	-25,3	16,71	3	y5
Clusterin	ELDESLQVAER.light	644,82	474,27	-25,3	y4	16,71	-25,3	16,71	3	y4
Clusterin	ELDESLQVAER.light	644,82	375,20	-25,3	y3	16,71	-25,3	16,71	3	y3
Clusterin	ELDESLQVAER.heavy	649,83	812,45	-25,3	y7	16,71	-25,3	16,71	3	y7
Clusterin	ELDESLQVAER.heavy	649,83	612,33	-25,3	y5	16,7	-25,3	16,7	3	y5
Clusterin	ELDESLQVAER.heavy	649,83	484,28	-25,3	y4	16,7	-25,3	16,7	3	y4
Clusterin	ELDESLQVAER.heavy	649,83	385,21	-25,3	y3	16,71	-25,3	16,71	3	y3
Clusterin	TLLSNLEEAK.light	559,31	215,14	-21,9	b2	22,15	-21,9	22,15	3	b2
Clusterin	TLLSNLEEAK.light	559,31	903,48	-21,9	y8	22,15	-21,9	22,15	3	y8
Clusterin	TLLSNLEEAK.light	559,31	790,39	-21,9	y7	22,15	-21,9	22,15	3	y7
Clusterin	TLLSNLEEAK.heavy	563,32	215,14	-21,9	b2	22,15	-21,9	22,15	3	b2
Clusterin	TLLSNLEEAK.heavy	563,32	911,49	-21,9	y8	22,15	-21,9	22,15	3	y8
Clusterin	TLLSNLEEAK.heavy	563,32	798,41	-21,9	y7	22,15	-21,9	22,15	3	y7
Coagulation_factor_X	TGIVSGFGR.light	447,25	272,16	-17,4	b3	16,58	-17,4	16,58	3	b3
Coagulation_factor_X	TGIVSGFGR.light	447,25	622,33	-17,4	y6	16,58	-17,4	16,58	3	y6
Coagulation_factor_X	TGIVSGFGR.light	447,25	523,26	-17,4	y5	16,57	-17,4	16,57	3	y5
Coagulation_factor_X	TGIVSGFGR.heavy	452,25	272,16	-17,4	b3	16,57	-17,4	16,57	3	b3
Coagulation_factor_X	TGIVSGFGR.heavy	452,25	632,34	-17,4	y6	16,58	-17,4	16,58	3	y6
Coagulation_factor_X	TGIVSGFGR.heavy	452,25	533,27	-17,4	y5	16,57	-17,4	16,57	3	y5
Complement_C1q_subcomponent_subunit_B	IAFSATR.light	383,22	652,34	-14,8	y6	#N/A	-14,8	#N/A	3	y6
Complement_C1q_subcomponent_subunit_B	IAFSATR.light	383,22	581,30	-14,8	y5	#N/A	-14,8	#N/A	3	y5
Complement_C1q_subcomponent_subunit_B	IAFSATR.light	383,22	434,24	-14,8	y4	#N/A	-14,8	#N/A	3	y4
Complement_C1q_subcomponent_subunit_B	IAFSATR.heavy	388,22	662,35	-14,8	y6	8,67	-14,8	8,67	3	y6
Complement_C1q_subcomponent_subunit_B	IAFSATR.heavy	388,22	591,31	-14,8	y5	8,54	-14,8	8,54	3	y5
Complement_C1q_subcomponent_subunit_B	IAFSATR.heavy	388,22	444,24	-14,8	y4	8,54	-14,8	8,54	3	y4
Complement_C1q_subcomponent_subunit_C	FQSVFTVTR.light	542,79	809,45	-21,2	y7	21,79	-21,2	21,79	3	y7
Complement_C1q_subcomponent_subunit_C	FQSVFTVTR.light	542,79	623,35	-21,2	y5	21,79	-21,2	21,79	3	y5
Complement_C1q_subcomponent_subunit_C	FQSVFTVTR.light	542,79	476,28	-21,2	y4	21,79	-21,2	21,79	3	y4

Complement_C1q_subcomponent_subunit_C	FQSVFTVTR.heavy	547,80	819,46	-21,2	y7	21,79	-21,2	21,79	3	y7
Complement_C1q_subcomponent_subunit_C	FQSVFTVTR.heavy	547,80	633,36	-21,2	y5	21,79	-21,2	21,79	3	y5
Complement_C1q_subcomponent_subunit_C	FQSVFTVTR.heavy	547,80	486,29	-21,2	y4	21,81	-21,2	21,81	3	y4
Complement_C1r_subcomponent	TNFDNDIALVR.light	639,33	571,39	-25,1	y5	22,85	-25,1	22,85	3	y5
Complement_C1r_subcomponent	TNFDNDIALVR.light	639,33	458,31	-25,1	y4	22,85	-25,1	22,85	3	y4
Complement_C1r_subcomponent	TNFDNDIALVR.light	639,33	387,27	-25,1	y3	22,85	-25,1	22,85	3	y3
Complement_C1r_subcomponent	TNFDNDIALVR.heavy	644,33	581,40	-25,1	y5	22,85	-25,1	22,85	3	y5
Complement_C1r_subcomponent	TNFDNDIALVR.heavy	644,33	468,32	-25,1	y4	22,85	-25,1	22,85	3	y4
Complement_C1r_subcomponent	TNFDNDIALVR.heavy	644,33	397,28	-25,1	y3	22,85	-25,1	22,85	3	y3
Complement_C1r_subcomponent	YTTEIIK.light	434,24	704,42	-16,9	y6	13,17	-16,9	13,17	3	y6
Complement_C1r_subcomponent	YTTEIIK.light	434,24	603,37	-16,9	y5	13,17	-16,9	13,17	3	y5
Complement_C1r_subcomponent	YTTEIIK.light	434,24	373,28	-16,9	y3	13,17	-16,9	13,17	3	y3
Complement_C1r_subcomponent	YTTEIIK.heavy	437,25	710,44	-16,9	y6	13,18	-16,9	13,18	3	y6
Complement_C1r_subcomponent	YTTEIIK.heavy	437,25	609,39	-16,9	y5	13,17	-16,9	13,17	3	y5
Complement_C1r_subcomponent	YTTEIIK.heavy	437,25	379,30	-16,9	y3	13,18	-16,9	13,18	3	y3
Complement_C2	HAFILQDTK.light	358,20	356,17	-12,4	b3	14,16	-12,4	14,16	3	b3
Complement_C2	HAFILQDTK.light	358,20	469,26	-12,4	b4	14,16	-12,4	14,16	3	b4
Complement_C2	HAFILQDTK.light	358,20	491,25	-12,4	y4	14,16	-12,4	14,16	3	y4
Complement_C2	HAFILQDTK.heavy	360,87	356,17	-12,4	b3	14,16	-12,4	14,16	3	b3
Complement_C2	HAFILQDTK.heavy	360,87	469,26	-12,4	b4	14,16	-12,4	14,16	3	b4
Complement_C2	HAFILQDTK.heavy	360,87	499,26	-12,4	y4	14,16	-12,4	14,16	3	y4
Complement_C2	TAVDHIR.light	406,22	639,36	-15,7	y5	4,96	-15,7	4,96	3	y5
Complement_C2	TAVDHIR.light	406,22	540,29	-15,7	y4	4,96	-15,7	4,96	3	y4
Complement_C2	TAVDHIR.light	406,22	425,26	-15,7	y3	4,96	-15,7	4,96	3	y3
Complement_C2	TAVDHIR.heavy	411,23	649,37	-15,7	y5	4,96	-15,7	4,96	3	y5
Complement_C2	TAVDHIR.heavy	411,23	550,30	-15,7	y4	4,96	-15,7	4,96	3	y4
Complement_C2	TAVDHIR.heavy	411,23	435,27	-15,7	y3	4,96	-15,7	4,96	3	y3
Complement_C3	TGLQEVEVK.light	501,78	731,39	-19,6	y6	13,69	-19,6	13,69	3	y6
Complement_C3	TGLQEVEVK.light	501,78	603,33	-19,6	y5	13,69	-19,6	13,69	3	y5
Complement_C3	TGLQEVEVK.light	501,78	422,74	-19,6	y7	13,69	-19,6	13,69	3	y7
Complement_C3	TGLQEVEVK.heavy	505,78	739,41	-19,6	y6	13,69	-19,6	13,69	3	y6
Complement_C3	TGLQEVEVK.heavy	505,78	611,35	-19,6	y5	13,69	-19,6	13,69	3	y5

Complement_C3	TGLQEVEVK.heavy	505,78	426,75	-19,6	y7	13,68	-19,6	13,68	3	y7
Complement_C3	SGIPIVTSPYQIHFTK.light	596,66	765,92	-21,2	y13	31,05	-21,2	31,05	3	y13
Complement_C3	SGIPIVTSPYQIHFTK.light	596,66	510,95	-21,2	y13	31,06	-21,2	31,06	3	y13
Complement_C3	SGIPIVTSPYQIHFTK.light	596,66	258,14	-21,2	b3	31,06	-21,2	31,06	3	b3
Complement_C3	SGIPIVTSPYQIHFTK.heavy	599,33	769,93	-21,2	y13	31,05	-21,2	31,05	3	y13
Complement_C3	SGIPIVTSPYQIHFTK.heavy	599,33	513,62	-21,2	y13	31,05	-21,2	31,05	3	y13
Complement_C3	SGIPIVTSPYQIHFTK.heavy	599,33	258,14	-21,2	b3	31,05	-21,2	31,05	3	b3
Complement_C4-B	GSFEFPVGDAVSK.light	670,33	568,24	-26,3	b5	25,33	-26,3	25,33	3	b5
Complement_C4-B	GSFEFPVGDAVSK.light	670,33	919,49	-26,3	y9	25,33	-26,3	25,33	3	y9
Complement_C4-B	GSFEFPVGDAVSK.light	670,33	772,42	-26,3	y8	25,32	-26,3	25,32	3	y8
Complement_C4-B	GSFEFPVGDAVSK.heavy	674,34	568,24	-26,3	b5	25,33	-26,3	25,33	3	b5
Complement_C4-B	GSFEFPVGDAVSK.heavy	674,34	927,50	-26,3	y9	25,32	-26,3	25,32	3	y9
Complement_C4-B	GSFEFPVGDAVSK.heavy	674,34	780,43	-26,3	y8	25,3	-26,3	25,3	3	y8
Complement_C4-B	VGDTLNLNLR.light	557,81	272,12	-21,8	b3	21,6	-21,8	21,6	3	b3
Complement_C4-B	VGDTLNLNLR.light	557,81	629,37	-21,8	y5	21,6	-21,8	21,6	3	y5
Complement_C4-B	VGDTLNLNLR.light	557,81	402,25	-21,8	y3	21,6	-21,8	21,6	3	y3
Complement_C4-B	VGDTLNLNLR.heavy	562,82	272,12	-21,8	b3	21,6	-21,8	21,6	3	b3
Complement_C4-B	VGDTLNLNLR.heavy	562,82	639,38	-21,8	y5	21,6	-21,8	21,6	3	y5
Complement_C4-B	VGDTLNLNLR.heavy	562,82	412,25	-21,8	y3	21,6	-21,8	21,6	3	y3
Coagulation_factor_XII	VVGGLVALR.light	442,29	784,50	-17,2	y8	20,82	-17,2	20,82	3	y8
Coagulation_factor_XII	VVGGLVALR.light	442,29	685,44	-17,2	y7	20,81	-17,2	20,81	3	y7
Coagulation_factor_XII	VVGGLVALR.light	442,29	458,31	-17,2	y4	20,81	-17,2	20,81	3	y4
Coagulation_factor_XII	VVGGLVALR.heavy	447,29	794,51	-17,2	y8	20,81	-17,2	20,81	3	y8
Coagulation_factor_XII	VVGGLVALR.heavy	447,29	695,44	-17,2	y7	20,81	-17,2	20,81	3	y7
Coagulation_factor_XII	VVGGLVALR.heavy	447,29	468,32	-17,2	y4	20,81	-17,2	20,81	3	y4
Coagulation_factor_XII	TEQAAVAR.light	423,23	231,10	-16,4	b2	3,38	-16,4	3,38	3	b2
Coagulation_factor_XII	TEQAAVAR.light	423,23	615,36	-16,4	y6	3,38	-16,4	3,38	3	y6
Coagulation_factor_XII	TEQAAVAR.light	423,23	487,30	-16,4	y5	3,38	-16,4	3,38	3	y5
Coagulation_factor_XII	TEQAAVAR.heavy	428,23	231,10	-16,4	b2	3,38	-16,4	3,38	3	b2
Coagulation_factor_XII	TEQAAVAR.heavy	428,23	625,37	-16,4	y6	3,38	-16,4	3,38	3	y6
Coagulation_factor_XII	TEQAAVAR.heavy	428,23	497,31	-16,4	y5	3,38	-16,4	3,38	3	y5
Complement_C5	VFQFLEK.light	455,76	811,43	-17,7	y6	23,54	-17,7	23,54	3	y6

Complement_C5	VFQFLEK.light	455,76	664,37	-17,7	y5	23,54	-17,7	23,54	3	y5
Complement_C5	VFQFLEK.light	455,76	247,14	-17,7	b2	23,54	-17,7	23,54	3	b2
Complement_C5	VFQFLEK.heavy	459,76	819,45	-17,7	y6	23,54	-17,7	23,54	3	y6
Complement_C5	VFQFLEK.heavy	459,76	672,38	-17,7	y5	23,52	-17,7	23,52	3	y5
Complement_C5	VFQFLEK.heavy	459,76	247,14	-17,7	b2	23,52	-17,7	23,52	3	b2
Complement_component_C8_beta_chain	SDLEVAHYK.light	354,51	203,07	-12,3	b2	10,21	-12,3	10,21	3	b2
Complement_component_C8_beta_chain	SDLEVAHYK.light	354,51	487,75	-12,3	y8	10,21	-12,3	10,21	3	y8
Complement_component_C8_beta_chain	SDLEVAHYK.light	354,51	430,24	-12,3	y7	10,2	-12,3	10,2	3	y7
Complement_component_C8_beta_chain	SDLEVAHYK.heavy	357,18	203,07	-12,3	b2	10,2	-12,3	10,2	3	b2
Complement_component_C8_beta_chain	SDLEVAHYK.heavy	357,18	491,76	-12,3	y8	10,2	-12,3	10,2	3	y8
Complement_component_C8_beta_chain	SDLEVAHYK.heavy	357,18	434,24	-12,3	y7	10,2	-12,3	10,2	3	y7
Complement_component_C9	LSPIYNLVPVK.light	621,88	442,30	-24,4	y4	34,26	-24,4	34,26	3	y4
Complement_component_C9	LSPIYNLVPVK.light	621,88	343,23	-24,4	y3	34,28	-24,4	34,28	3	y3
Complement_component_C9	LSPIYNLVPVK.light	621,88	521,82	-24,4	y9	34,23	-24,4	34,23	3	y9
Complement_component_C9	LSPIYNLVPVK.heavy	625,88	450,32	-24,4	y4	34,4	-24,4	34,4	3	y4
Complement_component_C9	LSPIYNLVPVK.heavy	625,88	351,25	-24,4	y3	34,38	-24,4	34,38	3	y3
Complement_component_C9	LSPIYNLVPVK.heavy	625,88	525,83	-24,4	y9	34,4	-24,4	34,4	3	y9
Complement_component_C9	VVEESELAR.light	516,27	328,19	-20,1	b3	9,73	-20,1	9,73	3	b3
Complement_component_C9	VVEESELAR.light	516,27	833,40	-20,1	y7	9,73	-20,1	9,73	3	y7
Complement_component_C9	VVEESELAR.light	516,27	575,31	-20,1	y5	9,63	-20,1	9,63	3	y5
Complement_component_C9	VVEESELAR.heavy	521,28	328,19	-20,1	b3	9,73	-20,1	9,73	3	b3
Complement_component_C9	VVEESELAR.heavy	521,28	843,41	-20,1	y7	9,73	-20,1	9,73	3	y7
Complement_component_C9	VVEESELAR.heavy	521,28	585,32	-20,1	y5	9,73	-20,1	9,73	3	y5
Complement_factor_D	THHDGAITER.light	379,52	239,11	-13,2	b2	2,33	-13,2	2,33	3	b2
Complement_factor_D	THHDGAITER.light	379,52	619,26	-13,2	b6	2,33	-13,2	2,33	3	b6
Complement_factor_D	THHDGAITER.light	379,52	405,21	-13,2	y3	2,33	-13,2	2,33	3	y3
Complement_factor_D	THHDGAITER.heavy	382,86	239,11	-13,2	b2	2,33	-13,2	2,33	3	b2
Complement_factor_D	THHDGAITER.heavy	382,86	619,26	-13,2	b6	2,33	-13,2	2,33	3	b6
Complement_factor_D	THHDGAITER.heavy	382,86	415,22	-13,2	y3	2,33	-13,2	2,33	3	y3
Complement_factor_I	HGNTDSEGIVEVK.light	462,23	525,21	-16,3	b5	10,31	-16,3	10,31	3	b5
Complement_factor_I	HGNTDSEGIVEVK.light	462,23	644,40	-16,3	y6	10,32	-16,3	10,32	3	y6
Complement_factor_I	HGNTDSEGIVEVK.light	462,23	474,29	-16,3	y4	10,31	-16,3	10,31	3	y4

Complement_factor_I	HGNTDSEGIVEVK.heavy	464,23	525,21	-16,3	b5	10,31	-16,3	10,31	3	b5
Complement_factor_I	HGNTDSEGIVEVK.heavy	464,23	650,42	-16,3	y6	10,32	-16,3	10,32	3	y6
Complement_factor_I	HGNTDSEGIVEVK.heavy	464,23	480,31	-16,3	y4	10,31	-16,3	10,31	3	y4
Complement_factor_I	TSSSFEVR.light	456,72	724,36	-17,8	y6	8,7	-17,8	8,7	3	y6
Complement_factor_I	TSSSFEVR.light	456,72	637,33	-17,8	y5	8,72	-17,8	8,72	3	y5
Complement_factor_I	TSSSFEVR.light	456,72	550,30	-17,8	y4	8,72	-17,8	8,72	3	y4
Complement_factor_I	TSSSFEVR.heavy	461,73	734,37	-17,8	y6	8,7	-17,8	8,7	3	y6
Complement_factor_I	TSSSFEVR.heavy	461,73	647,34	-17,8	y5	8,72	-17,8	8,72	3	y5
Complement_factor_I	TSSSFEVR.heavy	461,73	560,31	-17,8	y4	8,73	-17,8	8,73	3	y4
Corticosteroid-binding_globulin	ITQDAQLK.light	458,76	215,14	-17,8	b2	6,49	-17,8	6,49	3	b2
Corticosteroid-binding_globulin	ITQDAQLK.light	458,76	803,43	-17,8	y7	6,49	-17,8	6,49	3	y7
Corticosteroid-binding_globulin	ITQDAQLK.light	458,76	702,38	-17,8	y6	6,49	-17,8	6,49	3	y6
Corticosteroid-binding_globulin	ITQDAQLK.heavy	462,77	215,14	-17,8	b2	6,49	-17,8	6,49	3	b2
Corticosteroid-binding_globulin	ITQDAQLK.heavy	462,77	811,44	-17,8	y7	6,49	-17,8	6,49	3	y7
Corticosteroid-binding_globulin	ITQDAQLK.heavy	462,77	710,39	-17,8	y6	6,48	-17,8	6,48	3	y6
Fibrinogen_alpha_chain	GSESGIFTNTK.light	570,78	780,43	-22,3	y7	12,09	-22,3	12,09	3	y7
Fibrinogen_alpha_chain	GSESGIFTNTK.light	570,78	610,32	-22,3	y5	12,09	-22,3	12,09	3	y5
Fibrinogen_alpha_chain	GSESGIFTNTK.light	570,78	498,75	-22,3	y9	12,09	-22,3	12,09	3	y9
Fibrinogen_alpha_chain	GSESGIFTNTK.heavy	574,79	788,44	-22,3	y7	12,09	-22,3	12,09	3	y7
Fibrinogen_alpha_chain	GSESGIFTNTK.heavy	574,79	618,33	-22,3	y5	12,09	-22,3	12,09	3	y5
Fibrinogen_alpha_chain	GSESGIFTNTK.heavy	574,79	471,27	-22,3	y4	12,09	-22,3	12,09	3	y4
Fibrinogen_alpha_chain	GSESGIFTNTK.heavy	574,79	502,76	-22,3	y9	12,09	-22,3	12,09	3	y9
Fibrinogen_alpha_chain	ESSSHHPGIAEFPSR.light	546,59	506,27	-19,4	y4	12,33	-19,4	12,33	3	y4
Fibrinogen_alpha_chain	ESSSHHPGIAEFPSR.light	546,59	359,20	-19,4	y3	12,33	-19,4	12,33	3	y3
Fibrinogen_alpha_chain	ESSSHHPGIAEFPSR.light	546,59	487,26	-19,4	y9	12,33	-19,4	12,33	3	y9
Fibrinogen_alpha_chain	ESSSHHPGIAEFPSR.heavy	549,93	516,28	-19,4	y4	12,31	-19,4	12,31	3	y4
Fibrinogen_alpha_chain	ESSSHHPGIAEFPSR.heavy	549,93	369,21	-19,4	y3	12,33	-19,4	12,33	3	y3
Fibrinogen_alpha_chain	ESSSHHPGIAEFPSR.heavy	549,93	492,26	-19,4	y9	12,31	-19,4	12,31	3	y9
Fibulin-1	GYHLNEEGTR.light	392,52	422,19	-13,7	b7	6,61	-13,7	6,61	3	b7
Fibulin-1	GYHLNEEGTR.light	392,52	462,23	-13,7	y4	6,61	-13,7	6,61	3	y4
Fibulin-1	GYHLNEEGTR.light	392,52	333,19	-13,7	y3	6,61	-13,7	6,61	3	y3
Fibulin-1	GYHLNEEGTR.heavy	395,86	422,19	-13,7	b7	6,61	-13,7	6,61	3	b7

Fibulin-1	GYHLNEEGTR.heavy	395,86	472,24	-13,7	y4	6,61	-13,7	6,61	3	y4
Fibulin-1	GYHLNEEGTR.heavy	395,86	343,20	-13,7	y3	6,61	-13,7	6,61	3	y3
Fibulin-1	TGYYFDGISR.light	589,78	322,14	-23,1	b3	20,24	-23,1	20,24	3	b3
Fibulin-1	TGYYFDGISR.light	589,78	857,42	-23,1	y7	20,25	-23,1	20,25	3	y7
Fibulin-1	TGYYFDGISR.light	589,78	694,35	-23,1	y6	20,24	-23,1	20,24	3	y6
Fibulin-1	TGYYFDGISR.heavy	594,78	322,14	-23,1	b3	20,25	-23,1	20,25	3	b3
Fibulin-1	TGYYFDGISR.heavy	594,78	867,42	-23,1	y7	20,24	-23,1	20,24	3	y7
Fibulin-1	TGYYFDGISR.heavy	594,78	704,36	-23,1	y6	20,24	-23,1	20,24	3	y6
Gelsolin	AGALNSNDAFVLK.light	660,35	200,10	-25,9	b3	22,11	-25,9	22,11	3	b3
Gelsolin	AGALNSNDAFVLK.light	660,35	313,19	-25,9	b4	22,11	-25,9	22,11	3	b4
Gelsolin	AGALNSNDAFVLK.light	660,35	260,20	-25,9	y2	22,11	-25,9	22,11	3	y2
Gelsolin	AGALNSNDAFVLK.heavy	664,36	200,10	-25,9	b3	22,11	-25,9	22,11	3	b3
Gelsolin	AGALNSNDAFVLK.heavy	664,36	313,19	-25,9	b4	22,09	-25,9	22,09	3	b4
Gelsolin	AGALNSNDAFVLK.heavy	664,36	268,21	-25,9	y2	22,13	-25,9	22,13	3	y2
Gelsolin	TASDFITK.light	441,73	781,41	-17,2	y7	11,6	-17,2	11,6	3	y7
Gelsolin	TASDFITK.light	441,73	710,37	-17,2	y6	11,6	-17,2	11,6	3	y6
Gelsolin	TASDFITK.light	441,73	248,16	-17,2	y2	11,6	-17,2	11,6	3	y2
Gelsolin	TASDFITK.heavy	445,74	789,42	-17,2	y7	11,59	-17,2	11,59	3	y7
Gelsolin	TASDFITK.heavy	445,74	718,39	-17,2	y6	11,59	-17,2	11,59	3	y6
Gelsolin	TASDFITK.heavy	445,74	256,17	-17,2	y2	11,59	-17,2	11,59	3	y2
Haptoglobin	DYAEVGR.light	405,19	279,10	-15,7	b2	6,66	-15,7	6,66	3	b2
Haptoglobin	DYAEVGR.light	405,19	531,29	-15,7	y5	6,66	-15,7	6,66	3	y5
Haptoglobin	DYAEVGR.light	405,19	460,25	-15,7	y4	6,66	-15,7	6,66	3	y4
Haptoglobin	DYAEVGR.heavy	410,20	279,10	-15,7	b2	6,66	-15,7	6,66	3	b2
Haptoglobin	DYAEVGR.heavy	410,20	541,30	-15,7	y5	6,66	-15,7	6,66	3	y5
Haptoglobin	DYAEVGR.heavy	410,20	470,26	-15,7	y4	6,66	-15,7	6,66	3	y4
Haptoglobin	HYEGSTVPEK.light	573,77	301,13	-22,4	b2	4,45	-22,4	4,45	3	b2
Haptoglobin	HYEGSTVPEK.light	573,77	846,42	-22,4	y8	4,45	-22,4	4,45	3	y8
Haptoglobin	HYEGSTVPEK.light	573,77	373,21	-22,4	y3	4,45	-22,4	4,45	3	y3
Haptoglobin	HYEGSTVPEK.heavy	577,78	301,13	-22,4	b2	4,45	-22,4	4,45	3	b2
Haptoglobin	HYEGSTVPEK.heavy	577,78	854,43	-22,4	y8	4,45	-22,4	4,45	3	y8
Haptoglobin	HYEGSTVPEK.heavy	577,78	381,22	-22,4	y3	4,45	-22,4	4,45	3	y3

Hemoglobin_subunit_alpha	TYFPHFDLSHGSAQVK.light	611,97	711,86	-21,8	y13	23,15	-21,8	23,15	3	y13
Hemoglobin_subunit_alpha	TYFPHFDLSHGSAQVK.light	611,97	523,93	-21,8	y14	23,15	-21,8	23,15	3	y14
Hemoglobin_subunit_alpha	TYFPHFDLSHGSAQVK.light	611,97	442,56	-21,8	y12	23,15	-21,8	23,15	3	y12
Hemoglobin_subunit_alpha	TYFPHFDLSHGSAQVK.heavy	614,64	715,87	-21,8	y13	23,15	-21,8	23,15	3	y13
Hemoglobin_subunit_alpha	TYFPHFDLSHGSAQVK.heavy	614,64	526,60	-21,8	y14	23,16	-21,8	23,16	3	y14
Hemoglobin_subunit_alpha	TYFPHFDLSHGSAQVK.heavy	614,64	445,23	-21,8	y12	23,16	-21,8	23,16	3	y12
Hemoglobin_subunit_alpha	VGAHAGEYGAEALER.light	510,58	617,33	-18,1	y5	12,79	-18,1	12,79	3	y5
Hemoglobin_subunit_alpha	VGAHAGEYGAEALER.light	510,58	488,28	-18,1	y4	12,79	-18,1	12,79	3	y4
Hemoglobin_subunit_alpha	VGAHAGEYGAEALER.light	510,58	417,25	-18,1	y3	12,79	-18,1	12,79	3	y3
Hemoglobin_subunit_alpha	VGAHAGEYGAEALER.heavy	513,92	627,33	-18,1	y5	12,79	-18,1	12,79	3	y5
Hemoglobin_subunit_alpha	VGAHAGEYGAEALER.heavy	513,92	498,29	-18,1	y4	12,79	-18,1	12,79	3	y4
Hemoglobin_subunit_alpha	VGAHAGEYGAEALER.heavy	513,92	427,25	-18,1	y3	12,79	-18,1	12,79	3	y3
sp|P02790|HEMO_HUMAN	NFPSPVDAAFR.light	610,81	959,49	-23,9	y9	25,39	-23,9	25,39	3	y9
sp|P02790|HEMO_HUMAN	NFPSPVDAAFR.light	610,81	775,41	-23,9	y7	25,38	-23,9	25,38	3	y7
sp|P02790|HEMO_HUMAN	NFPSPVDAAFR.light	610,81	464,26	-23,9	y4	25,39	-23,9	25,39	3	y4
sp|P02790|HEMO_HUMAN	NFPSPVDAAFR.light	610,81	480,25	-23,9	y9	25,37	-23,9	25,37	3	y9
sp|P02790|HEMO_HUMAN	NFPSPVDAAFR.heavy	615,81	969,50	-23,9	y9	25,36	-23,9	25,36	3	y9
sp|P02790|HEMO_HUMAN	NFPSPVDAAFR.heavy	615,81	785,42	-23,9	y7	25,38	-23,9	25,38	3	y7
sp|P02790|HEMO_HUMAN	NFPSPVDAAFR.heavy	615,81	474,27	-23,9	y4	25,38	-23,9	25,38	3	y4
sp|P02790|HEMO_HUMAN	NFPSPVDAAFR.heavy	615,81	485,26	-23,9	y9	25,39	-23,9	25,39	3	y9
Heparin_cofactor_2	TLEAQLTPR.light	514,79	814,44	-20,1	y7	15,43	-20,1	15,43	3	y7
Heparin_cofactor_2	TLEAQLTPR.light	514,79	685,40	-20,1	y6	15,43	-20,1	15,43	3	y6
Heparin_cofactor_2	TLEAQLTPR.light	514,79	373,22	-20,1	y3	15,43	-20,1	15,43	3	y3
Heparin_cofactor_2	TLEAQLTPR.light	514,79	344,18	-20,1	b3	15,43	-20,1	15,43	3	b3
Heparin_cofactor_2	TLEAQLTPR.heavy	519,79	824,45	-20,1	y7	15,43	-20,1	15,43	3	y7
Heparin_cofactor_2	TLEAQLTPR.heavy	519,79	695,41	-20,1	y6	15,43	-20,1	15,43	3	y6
Heparin_cofactor_2	TLEAQLTPR.heavy	519,79	383,23	-20,1	y3	15,43	-20,1	15,43	3	y3
Heparin_cofactor_2	TLEAQLTPR.heavy	519,79	344,18	-20,1	b3	15,44	-20,1	15,44	3	b3
Heparin_cofactor_2	SVNDLYIQK.light	540,29	187,11	-21,1	b2	15,05	-21,1	15,05	3	b2
Heparin_cofactor_2	SVNDLYIQK.light	540,29	893,47	-21,1	y7	15,05	-21,1	15,05	3	y7
Heparin_cofactor_2	SVNDLYIQK.light	540,29	551,32	-21,1	y4	15,05	-21,1	15,05	3	y4
Heparin_cofactor_2	SVNDLYIQK.heavy	544,30	187,11	-21,1	b2	15,05	-21,1	15,05	3	b2

Heparin_cofactor_2	SVNDLYIQK.heavy	544,30	901,49	-21,1	y7	15,04	-21,1	15,04	3	y7
Heparin_cofactor_2	SVNDLYIQK.heavy	544,30	559,33	-21,1	y4	15,04	-21,1	15,04	3	y4
Insulin-like_growth_factor_binding_protein_acid_labile_subunit	LAELPADALGPLQR.light	732,41	314,17	-28,8	b3	29,55	-28,8	29,55	3	b3
Insulin-like_growth_factor_binding_protein_acid_labile_subunit	LAELPADALGPLQR.light	732,41	427,26	-28,8	b4	29,55	-28,8	29,55	3	b4
Insulin-like_growth_factor_binding_protein_acid_labile_subunit	LAELPADALGPLQR.light	732,41	519,29	-28,8	y10	29,55	-28,8	29,55	3	y10
Insulin-like_growth_factor_binding_protein_acid_labile_subunit	LAELPADALGPLQR.heavy	737,42	524,29	-28,8	y10	29,55	-28,8	29,55	3	y10
Insulin-like_growth_factor_binding_protein_acid_labile_subunit	LAELPADALGPLQR.heavy	737,42	314,17	-28,8	b3	29,55	-28,8	29,55	3	b3
Insulin-like_growth_factor_binding_protein_acid_labile_subunit	LAELPADALGPLQR.heavy	737,42	427,26	-28,8	b4	29,54	-28,8	29,54	3	b4
Insulin-like_growth_factor-binding_protein_3	FLNVLSPR.light	473,28	685,40	-18,4	y6	23,93	-18,4	23,93	3	y6
Insulin-like_growth_factor-binding_protein_3	FLNVLSPR.light	473,28	472,29	-18,4	y4	23,94	-18,4	23,94	3	y4
Insulin-like_growth_factor-binding_protein_3	FLNVLSPR.light	473,28	359,20	-18,4	y3	23,94	-18,4	23,94	3	y3
Insulin-like_growth_factor-binding_protein_3	FLNVLSPR.heavy	478,28	695,41	-18,4	y6	23,94	-18,4	23,94	3	y6
Insulin-like_growth_factor-binding_protein_3	FLNVLSPR.heavy	478,28	482,30	-18,4	y4	23,94	-18,4	23,94	3	y4
Insulin-like_growth_factor-binding_protein_3	FLNVLSPR.heavy	478,28	369,21	-18,4	y3	23,95	-18,4	23,95	3	y3
Inter-alpha-trypsin_inhibitor_heavy_chain_H2	FYNQVSTPLLR.light	669,36	686,42	-26,3	y6	24,16	-26,3	24,16	3	y6
Inter-alpha-trypsin_inhibitor_heavy_chain_H2	FYNQVSTPLLR.light	669,36	498,34	-26,3	y4	24,16	-26,3	24,16	3	y4
Inter-alpha-trypsin_inhibitor_heavy_chain_H2	FYNQVSTPLLR.light	669,36	514,30	-26,3	y9	24,16	-26,3	24,16	3	y9
Inter-alpha-trypsin_inhibitor_heavy_chain_H2	FYNQVSTPLLR.heavy	674,37	696,43	-26,3	y6	24,15	-26,3	24,15	3	y6
Inter-alpha-trypsin_inhibitor_heavy_chain_H2	FYNQVSTPLLR.heavy	674,37	508,35	-26,3	y4	24,16	-26,3	24,16	3	y4
Inter-alpha-trypsin_inhibitor_heavy_chain_H2	FYNQVSTPLLR.heavy	674,37	519,30	-26,3	y9	24,16	-26,3	24,16	3	y9
Inter-alpha-trypsin_inhibitor_heavy_chain_H2	SLAPTAAAK.light	415,24	272,16	-16,1	b3	7,36	-16,1	7,36	3	b3
Inter-alpha-trypsin_inhibitor_heavy_chain_H2	SLAPTAAAK.light	415,24	629,36	-16,1	y7	7,36	-16,1	7,36	3	y7
Inter-alpha-trypsin_inhibitor_heavy_chain_H2	SLAPTAAAK.light	415,24	558,32	-16,1	y6	7,36	-16,1	7,36	3	y6
Inter-alpha-trypsin_inhibitor_heavy_chain_H2	SLAPTAAAK.heavy	419,25	272,16	-16,1	b3	7,36	-16,1	7,36	3	b3
Inter-alpha-trypsin_inhibitor_heavy_chain_H2	SLAPTAAAK.heavy	419,25	637,38	-16,1	y7	7,36	-16,1	7,36	3	y7
Inter-alpha-trypsin_inhibitor_heavy_chain_H2	SLAPTAAAK.heavy	419,25	566,34	-16,1	y6	7,36	-16,1	7,36	3	y6
Lipopolysaccharide-binding_protein	ITLPDFTGDLR.light	624,34	920,45	-24,5	y8	33,06	-24,5	33,06	3	y8
Lipopolysaccharide-binding_protein	ITLPDFTGDLR.light	624,34	517,27	-24,5	y9	33,06	-24,5	33,06	3	y9
Lipopolysaccharide-binding_protein	ITLPDFTGDLR.light	624,34	460,73	-24,5	y8	33,07	-24,5	33,07	3	y8

Lipopolysaccharide-binding_protein	ITLPDFTGDLR.heavy	629,34	930,46	-24,5	y8	33,06	-24,5	33,06	3	y8
Lipopolysaccharide-binding_protein	ITLPDFTGDLR.heavy	629,34	522,27	-24,5	y9	33,07	-24,5	33,07	3	y9
Lipopolysaccharide-binding_protein	ITLPDFTGDLR.heavy	629,34	465,73	-24,5	y8	33,06	-24,5	33,06	3	y8
Lumican	FNALQYLR.light	512,78	262,12	-20	b2	27,2	-20	27,2	3	b2
Lumican	FNALQYLR.light	512,78	763,45	-20	y6	27,21	-20	27,21	3	y6
Lumican	FNALQYLR.light	512,78	579,32	-20	y4	27,21	-20	27,21	3	y4
Lumican	FNALQYLR.heavy	517,79	262,12	-20	b2	27,2	-20	27,2	3	b2
Lumican	FNALQYLR.heavy	517,79	773,45	-20	y6	27,21	-20	27,21	3	y6
Lumican	FNALQYLR.heavy	517,79	589,33	-20	y4	27,21	-20	27,21	3	y4
Neuropilin-2	ALQVVR.light	343,22	313,19	-13,2	b3	9,12	-13,2	9,12	3	b3
Neuropilin-2	ALQVVR.light	343,22	501,31	-13,2	y4	9,11	-13,2	9,11	3	y4
Neuropilin-2	ALQVVR.light	343,22	373,26	-13,2	y3	9,12	-13,2	9,12	3	y3
Neuropilin-2	ALQVVR.heavy	348,23	313,19	-13,2	b3	9,11	-13,2	9,11	3	b3
Neuropilin-2	ALQVVR.heavy	348,23	511,32	-13,2	y4	9,12	-13,2	9,12	3	y4
Neuropilin-2	ALQVVR.heavy	348,23	383,26	-13,2	y3	9,11	-13,2	9,11	3	y3
PEDF	TVQAVLTVPK.light	528,33	329,18	-20,6	b3	19,63	-20,6	19,63	3	b3
PEDF	TVQAVLTVPK.light	528,33	727,47	-20,6	y7	19,63	-20,6	19,63	3	y7
PEDF	TVQAVLTVPK.light	528,33	557,37	-20,6	y5	19,63	-20,6	19,63	3	y5
PEDF	TVQAVLTVPK.heavy	532,33	329,18	-20,6	b3	19,63	-20,6	19,63	3	b3
PEDF	TVQAVLTVPK.heavy	532,33	735,49	-20,6	y7	19,63	-20,6	19,63	3	y7
PEDF	TVQAVLTVPK.heavy	532,33	565,38	-20,6	y5	19,63	-20,6	19,63	3	y5
sp|P02753|RET4_HUMAN	YWGVASFLQK.light	599,82	849,48	-23,5	y8	33,14	-23,5	33,14	3	y8
sp|P02753|RET4_HUMAN	YWGVASFLQK.light	599,82	693,39	-23,5	y6	33,14	-23,5	33,14	3	y6
sp|P02753|RET4_HUMAN	YWGVASFLQK.light	599,82	407,17	-23,5	b3	33,14	-23,5	33,14	3	b3
sp|P02753|RET4_HUMAN	YWGVASFLQK.heavy	603,82	857,50	-23,5	y8	33,14	-23,5	33,14	3	y8
sp|P02753|RET4_HUMAN	YWGVASFLQK.heavy	603,82	701,41	-23,5	y6	33,15	-23,5	33,15	3	y6
sp|P02753|RET4_HUMAN	YWGVASFLQK.heavy	603,82	407,17	-23,5	b3	33,12	-23,5	33,12	3	b3
Protein_AMBP	ETLLQDFR.light	511,27	678,36	-19,9	y5	23,4	-19,9	23,4	3	y5
Protein_AMBP	ETLLQDFR.light	511,27	565,27	-19,9	y4	23,4	-19,9	23,4	3	y4
Protein_AMBP	ETLLQDFR.light	511,27	437,21	-19,9	y3	23,4	-19,9	23,4	3	y3
Protein_AMBP	ETLLQDFR.heavy	516,27	688,37	-19,9	y5	23,4	-19,9	23,4	3	y5
Protein_AMBP	ETLLQDFR.heavy	516,27	575,28	-19,9	y4	23,4	-19,9	23,4	3	y4

Protein_AMBP	ETLLQDFR.heavy	516,27	447,22	-19,9	y3	23,4	-19,9	23,4	3	y3
Protein_AMBP	HHGPTITAK.light	481,26	687,40	-18,7	y7	3,12	-18,7	3,12	3	y7
Protein_AMBP	HHGPTITAK.light	481,26	319,20	-18,7	y3	3,12	-18,7	3,12	3	y3
Protein_AMBP	HHGPTITAK.light	481,26	412,73	-18,7	y8	3,11	-18,7	3,11	3	y8
Protein_AMBP	HHGPTITAK.heavy	485,27	695,42	-18,7	y7	3,12	-18,7	3,12	3	y7
Protein_AMBP	HHGPTITAK.heavy	485,27	327,21	-18,7	y3	3,12	-18,7	3,12	3	y3
Protein_AMBP	HHGPTITAK.heavy	485,27	416,74	-18,7	y8	3,11	-18,7	3,11	3	y8
Prothrombin	ELLESYIDGR.light	597,80	485,26	-23,4	b4	23,29	-23,4	23,29	3	b4
Prothrombin	ELLESYIDGR.light	597,80	710,35	-23,4	y6	23,29	-23,4	23,29	3	y6
Prothrombin	ELLESYIDGR.light	597,80	623,31	-23,4	y5	23,29	-23,4	23,29	3	y5
Prothrombin	ELLESYIDGR.heavy	602,81	485,26	-23,4	b4	23,29	-23,4	23,29	3	b4
Prothrombin	ELLESYIDGR.heavy	602,81	720,36	-23,4	y6	23,27	-23,4	23,27	3	y6
Prothrombin	ELLESYIDGR.heavy	602,81	633,32	-23,4	y5	23,29	-23,4	23,29	3	y5
Prothrombin	TATSEYQTFFNPR.light	781,37	1072,52	-30,7	y8	23,47	-30,7	23,47	3	y8
Prothrombin	TATSEYQTFFNPR.light	781,37	680,35	-30,7	y5	23,47	-30,7	23,47	3	y5
Prothrombin	TATSEYQTFFNPR.light	781,37	272,17	-30,7	y2	23,47	-30,7	23,47	3	y2
Prothrombin	TATSEYQTFFNPR.heavy	786,37	1082,53	-30,7	y8	23,45	-30,7	23,45	3	y8
Prothrombin	TATSEYQTFFNPR.heavy	786,37	690,36	-30,7	y5	23,45	-30,7	23,45	3	y5
Prothrombin	TATSEYQTFFNPR.heavy	786,37	282,18	-30,7	y2	23,47	-30,7	23,47	3	y2
Transferrin	DGAGDVAFVK.light	489,75	244,09	-19,1	b3	15,63	-19,1	15,63	3	b3
Transferrin	DGAGDVAFVK.light	489,75	735,40	-19,1	y7	15,63	-19,1	15,63	3	y7
Transferrin	DGAGDVAFVK.light	489,75	464,29	-19,1	y4	15,63	-19,1	15,63	3	y4
Transferrin	DGAGDVAFVK.heavy	493,76	244,09	-19,1	b3	15,63	-19,1	15,63	3	b3
Transferrin	DGAGDVAFVK.heavy	493,76	743,42	-19,1	y7	15,63	-19,1	15,63	3	y7
Transferrin	DGAGDVAFVK.heavy	493,76	472,30	-19,1	y4	15,63	-19,1	15,63	3	y4
Transferrin	EGYYGYTGAFR.light	642,29	350,13	-25,2	b3	19,57	-25,2	19,57	3	b3
Transferrin	EGYYGYTGAFR.light	642,29	771,38	-25,2	y7	19,56	-25,2	19,56	3	y7
Transferrin	EGYYGYTGAFR.light	642,29	551,29	-25,2	y5	19,57	-25,2	19,57	3	y5
Transferrin	EGYYGYTGAFR.heavy	647,29	350,13	-25,2	b3	19,55	-25,2	19,55	3	b3
Transferrin	EGYYGYTGAFR.heavy	647,29	781,39	-25,2	y7	19,56	-25,2	19,56	3	y7
Transferrin	EGYYGYTGAFR.heavy	647,29	561,30	-25,2	y5	19,56	-25,2	19,56	3	y5
Serum_amyloid_A-4_protein	GNYDAAQR.light	447,71	723,34	-17,4	y6	3,13	-17,4	3,13	3	y6

Serum_amyloid_A-4_protein	GNYDAAQR.light	447,71	445,25	-17,4	y4	3,13	-17,4	3,13	3	y4
Serum_amyloid_A-4_protein	GNYDAAQR.light	447,71	374,21	-17,4	y3	3,13	-17,4	3,13	3	y3
Serum_amyloid_A-4_protein	GNYDAAQR.heavy	452,71	733,35	-17,4	y6	3,13	-17,4	3,13	3	y6
Serum_amyloid_A-4_protein	GNYDAAQR.heavy	452,71	455,26	-17,4	y4	3,13	-17,4	3,13	3	y4
Serum_amyloid_A-4_protein	GNYDAAQR.heavy	452,71	384,22	-17,4	y3	3,13	-17,4	3,13	3	y3
Thyroxine-binding_globulin	AVLHIGEK.light	289,51	398,24	-9,9	y7	10,25	-9,9	10,25	3	y7
Thyroxine-binding_globulin	AVLHIGEK.light	289,51	348,71	-9,9	y6	10,21	-9,9	10,21	3	y6
Thyroxine-binding_globulin	AVLHIGEK.light	289,51	292,16	-9,9	y5	10,25	-9,9	10,25	3	y5
Thyroxine-binding_globulin	AVLHIGEK.heavy	292,18	402,25	-9,9	y7	10,21	-9,9	10,21	3	y7
Thyroxine-binding_globulin	AVLHIGEK.heavy	292,18	352,71	-9,9	y6	10,16	-9,9	10,16	3	y6
Thyroxine-binding_globulin	AVLHIGEK.heavy	292,18	296,17	-9,9	y5	10,16	-9,9	10,16	3	y5
Vitamin_D-binding_protein	THLPEVFLSK.light	390,89	494,30	-13,6	y4	21,68	-13,6	21,68	3	y4
Vitamin_D-binding_protein	THLPEVFLSK.light	390,89	347,23	-13,6	y3	21,68	-13,6	21,68	3	y3
Vitamin_D-binding_protein	THLPEVFLSK.light	390,89	352,20	-13,6	b3	21,7	-13,6	21,7	3	b3
Vitamin_D-binding_protein	THLPEVFLSK.light	390,89	578,29	-13,6	b5	21,7	-13,6	21,7	3	b5
Vitamin_D-binding_protein	THLPEVFLSK.heavy	393,56	502,31	-13,6	y4	21,68	-13,6	21,68	3	y4
Vitamin_D-binding_protein	THLPEVFLSK.heavy	393,56	355,24	-13,6	y3	21,68	-13,6	21,68	3	y3
Vitamin_D-binding_protein	THLPEVFLSK.heavy	393,56	352,20	-13,6	b3	21,68	-13,6	21,68	3	b3
Vitamin_D-binding_protein	THLPEVFLSK.heavy	393,56	578,29	-13,6	b5	21,68	-13,6	21,68	3	b5
Vitamin_D-binding_protein	ELPEHTVK.light	318,17	613,33	-10,9	y5	5,77	-10,9	5,77	3	y5
Vitamin_D-binding_protein	ELPEHTVK.light	318,17	355,70	-10,9	y6	5,77	-10,9	5,77	3	y6
Vitamin_D-binding_protein	ELPEHTVK.light	318,17	237,47	-10,9	y6	5,77	-10,9	5,77	3	y6
Vitamin_D-binding_protein	ELPEHTVK.heavy	320,85	621,34	-10,9	y5	5,77	-10,9	5,77	3	y5
Vitamin_D-binding_protein	ELPEHTVK.heavy	320,85	359,70	-10,9	y6	5,77	-10,9	5,77	3	y6
Vitamin_D-binding_protein	ELPEHTVK.heavy	320,85	240,14	-10,9	y6	5,77	-10,9	5,77	3	y6
Vitamin_K-dependent_protein_S	VYFAGFPR.light	478,75	694,37	-18,6	y6	23,84	-18,6	23,84	3	y6
Vitamin_K-dependent_protein_S	VYFAGFPR.light	478,75	547,30	-18,6	y5	23,84	-18,6	23,84	3	y5
Vitamin_K-dependent_protein_S	VYFAGFPR.light	478,75	476,26	-18,6	y4	23,84	-18,6	23,84	3	y4
Vitamin_K-dependent_protein_S	VYFAGFPR.heavy	483,76	704,38	-18,6	y6	23,83	-18,6	23,83	3	y6
Vitamin_K-dependent_protein_S	VYFAGFPR.heavy	483,76	557,31	-18,6	y5	23,83	-18,6	23,83	3	y5
Vitamin_K-dependent_protein_S	VYFAGFPR.heavy	483,76	486,27	-18,6	y4	23,83	-18,6	23,83	3	y4
Beta-2-glycoprotein 1	ATVVYQGER.light	511,77	850,44	-20	y7	7,9	-20	7,9	3	y7

Beta-2-glycoprotein 1	ATVVYQGER.light	511,77	751,37	-20	y6	7,9	-20	7,9	3	y6
Beta-2-glycoprotein 1	ATVVYQGER.light	511,77	652,30	-20	y5	7,9	-20	7,9	3	y5
Beta-2-glycoprotein 1	ATVVYQGER.light	511,77	662,35	-20	b6	7,9	-20	7,9	3	b6
Beta-2-glycoprotein 1	ATVVYQGER.heavy	516,77	860,45	-20	y7	7,9	-20	7,9	3	y7
Beta-2-glycoprotein 1	ATVVYQGER.heavy	516,77	761,38	-20	y6	7,9	-20	7,9	3	y6
Beta-2-glycoprotein 1	ATVVYQGER.heavy	516,77	662,31	-20	y5	7,9	-20	7,9	3	y5
Beta-2-glycoprotein 1	ATVVYQGER.heavy	516,77	662,35	-20	b6	7,9	-20	7,9	3	b6

Figure S1: LC-MRM chromatogram and calibration curves

Panel A: LC-MRM chromatogram showing many eluting peptides (diagrammed with different colors and a three code letters corresponding to the three first amino-acids) over the peptide elution range (between 1 and 37 minutes). On the y axis, MS intensities of all the peptides were reported.

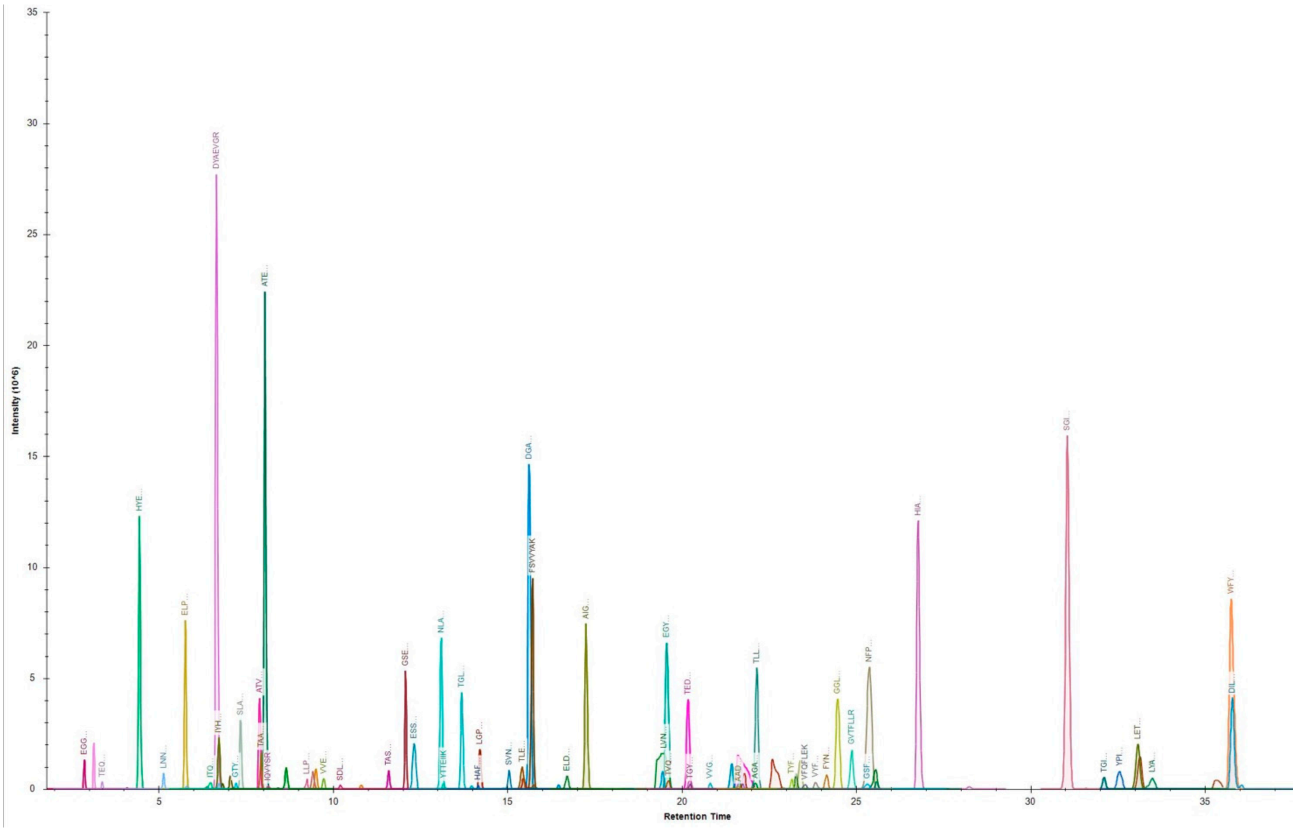

Sup Methods

Chemicals used

Serum (SST BD Vacutainer ® ref 368815) and plasma (EDTA K2 BD Vacutainer ® ref 367864) tubes.

DL-dithiothreitol (DTT), Iodoacetamide (IAA) from VWR (Radnor, USA)

AssayMAP Cartridge Reverse phase and C18 from Agilent technologies (Santa Clara, USA);

Acetonitrile ULC/MS, formic acid ULC/MS, Water ULC/MS from Biosolve (Dieuze, France);

Protein LoBind tubes 1.5 and 0.5 ml from Eppendorf (Hambourg, Germany).

Trizma® Hydrochloride (Tris-HCl) from Sigma Aldrich (Saint-Louis, USA);

Trypsin/LysC from Promega (Madison, USA)

Cryocheck^{TM} Normal Reference Plasma, from Cryopep (Montpellier, France)

TFN-Specimen Collection card included in a DBS collection kit (Ref Art002) prepared by SpotToLab® (France).

Preparation of samples before mass spectrometry analysis

The sample preparation protocols were automated using an Automatic puncher (PerkinElmer, CT USA) and an Agilent AssayMap Bravo Liquid Handling Platform (Agilent Technologies, Lexington, USA).

In all the sample, a 6-mm diameter punch was cut out of the cards using an automatized DBS Puncher instrument (1296-071, PerkinElmer, CT USA).

The punch was placed in a 96-deep-well plate (ABgene 1127, Portsmouth, USA). Protein content of the sample was extracted, reduced and alkylated, before a protein digestion with trypsin using a protocol patented (WO/2020/234287). All these steps were performed on BRAVO AssayMap automate (Agilent, Lexington, USA).

Briefly to DBS sample, proteins were eluted with 200uL ammonium bicarbonate (50mM), mixed at 1500rpm over 30min; denaturated by adding 200µL urea (8M) and shaken for 10 minutes (Sup Figure 1). Disulfide bridges were then reduced (21 µL of DTT 0.2M, 12 µL of Tris 1M pH 8.5) one hour at 37°C with stirring at 450rpm, and alkylated (18 µL of IAA 1M, 6 µL of Tris 1M pH 10) 30 minutes in the dark at 37°C with stirring at 450rpm. To stop the alkylation, 23µL DTT 0,2M was added. Sample was acidified with 10µL of formic acid and transfer into a new plate. Sample was cleaned on reverse phase tips (G5496-60086, Agilent T, Lexington, USA) following recommendation on the patent WO/2020/234287. Eluted samples were then dried on plate using a vacuum concentrator (Labconco, Kansas, USA). The tryptic digestion was carried out by resuspension of the sample with 2,8 µg of Trypsin/LysC and 20 µL of Tris 20 mM pH 8.5. The samples were incubated 14 hours at 37°C with shaking (450rpm). 3µL formic acid (pH<4) were added to stop the enzymatic reactions.

To the 2µL of plasma sample, sample preparation was also performed on BRAVO AssayMap automate (Agilent, Lexington, USA) with 96-well plate (PCR Eppendorf twin.tec® 96 LoBind, Eppendorf, Hambourg, Germany).

Normal Reference Plasma (Cryocheck^{TM}) was used as External quality control sample. This sample was analyzed in parallel in randomly select 8 positions in the 96-well plate. Inter-assay variability was calculated on these samples.

Briefly, plasma sample was denaturated with 100 µL of Urea 4,8 M in Tris 50 mM pH 8,5 and mixing at 450rpm during 30 minutes. Then 6µL of DTT 0.2M were added to perform the reduction. Thereafter the samples were incubated one hour at 37°C while shaking (450rpm). To alkylate the samples, 12µL of IAA 400mM in Tris 1M pH 10 were added. The samples were incubated 30 minutes at 37°C while shaking (450rpm). The tryptic digestion was carried out by a sample with 420 µL of DTT 2 mM, Tris 20 mM pH 8.5 followed by 18µL of Trypsin/LysC (0.31µg/µL). The samples were incubated 14 hours at 37°C while shaking (450rpm). 6µL formic acid (pH<4) were added to stop the enzymatic reactions. The tryptic peptides were desalted with C18 Tips conditioned with 50 µL of 70% ACN/0.1% FA and equilibrated with 50 µL of 0.1% FA. Loaded samples were washed twice with 50 µL of 0.1% FA and eluted with 50 µL of 70% ACN/0.1% FA. Elutions were dried by centrifugal evaporation on Speedvac (Labconco, Kansas, USA).

Mass Spectrometry Analysis

Before injection, samples were resuspended in 20 µL of 2% acetonitrile/0.1% formic acid/97.9% water and spiked with “Stable isotopically labelled peptide mix” described below. Samples were stirred for 10 min. Then they were centrifuged three minutes at 16000g. The liquid was transferred in polypropylene vials, ready for

analysis. All samples prepare were injected in duplicat. Between each measurement, a blank was measured to avoid carry-over.

LC separation of tryptic peptides was carried out on a UHPLC Nexera LC-40 liquid chromatography system (Shimadzu Corporation, Kyoto, Japan). Peptides were resolved using a reversed-phase column (Shim-pack GISSFTF-HP C18, 3 µm, 150× 2.1 mm) at a flow rate of 400 µl/min, and a temperature at 50°C.

A 48 min multistep gradient was performed using solvent B (99.9% acetonitrile and 0.1% formic acid), starting with 3% and increasing to 5% in 1.5 min, to 14% at 16 min, to 16% at 18 min, to 25% at 38 min. At 39 min, the column was flushed for 4 min with 80% of solvent B and then reconditioned with 3% of solvent B for 4 min.

Peptide analyses were carried out on a QqQ MS system (LCMS-8060, Shimadzu Corporation, Kyoto, Japan) in positive ion mode. The ESI source was set as follows: DL: 150°C, heat block: 250°C, interface: 400°C, nebulizer gas: 2 l/min, drying gas: 5 l/min, heating gas: 15 l/min. The Mass Spectrometer operated in scheduled MRM mode with a time windows of 3 minutes and a maximum loop time of 1.172s. Quadrupole resolution was unit to Q1 and Q3. CID gas pressure was fixed to 350 kPa. Collision energies (CE) were optimized for all the peptide transitions of interest (Sup Table 5). Finally, in our LC-MRM experiment 91 peptides were monitored corresponding to 66 proteins.

LabSolutions software (version 5.97) was used for data acquisition. Open-source Skyline® 20.2 software (https://skyline.ms/project/home) was used to analyse MRM data. The peak detection was performed automatically by the software and checked manually. Excel software was used to compute the ratio heavy/light.

Stable isotopically labelled peptide mix

PeptiQuant™ Biomarker Assessment Kit (BAK-76) from CIL (Cambridge Isotope Laboratories, Tewksbury, Massachusetts) (reference: BAK-A6495-76) was used to normalize the MS signal of 101 peptides corresponding to 76 proteins (see below). 4µL of the SIS (Stable Isotope Standards) peptide mixture were spiked to each sample.

Protein Name	Peptide	UniProt KB Accession No.	SIS added in ng/mL
Afamin	DADPDTFFAK	P43652	628,97
Alpha-1-antichymotrypsin	NLAVSQVVHK	P01011	9265,86
Alpha-1B-glycoprotein	GVTFLLR	P04217	4170,61
Alpha-1B-glycoprotein	LETPDFQLFK	P04217	11734,02
Alpha-2-antiplasmin	LGNQEPGGQTALK	P08697	3002,23
Alpha-2-HS-glycoprotein	FSVVYAK	P02765	13138,32
Alpha-2-macroglobulin	AIGYLNTGYQR	P01023	123951,41
Antithrombin-III	DDLYVSDAFHK	P01008	1144,96
Apolipoprotein A-I	ATEHLSTLSEK	P02647	123653,75
Apolipoprotein A-IV	LLPHANEVSQK	P06727	3684,45

Apolipoprotein B-100	TGISPLALIK	P04114	35440,35
Apolipoprotein C-II	TAAQNLYEK	P02655	110,45
Apolipoprotein D	VLNQELR	P05090	4334,67
Apolipoprotein E	AATVGSLAGQPLQER	P02649	2606,90
Apolipoprotein E	LGPLVEQGR	P02649	3525,83
Apolipoprotein M	AFLLTPR	O95445	4517,27
Apolipoprotein(a)	GTYSTTVTGR	P08519	12879,13
Beta-2-glycoprotein 1	ATVVYQGER	P02749	5836,99
Beta-2-microglobulin	IQVYSR	P61769	58,09
Beta-2-microglobulin	VNHVTLSQPK	P61769	166,59
Beta-Ala-His dipeptidase	SVVLIPLGAVDDGEHSQNEK	Q96KN2	162,47
C4b-binding protein alpha chain	EDVYVVGTVLR	P04003	840,14
C4b-binding protein alpha chain	LNNGEITQHR	P04003	1756,37
Carbonic anhydrase 1	VLDALQAIK	P00915	133,53
Cartilage acidic protein 1	GVASLFAGR	Q9NQ79	126,57
CD5 antigen-like	LVGGLHR	O43866	4058,08
cDNA FLJ53327	EGGQTAPASTR	B7Z2X4	8025,93
Ceruloplasmin	IYHSHIDAPK	P00450	207231,05
Cholinesterase	YLTLNTESTR	P06276	323,71
Clusterin	ELDESLQVAER	P10909	13476,30
Clusterin	TLLSNLEEAK	P10909	2102,63
Coagulation factor V	AEVDDVIQVR	P12259	1668,69
Coagulation factor X	TGIVSGFGR	P00742	945,78
Coagulation factor XI	TSESGLPSTR	P03951	491,16
Coagulation factor XII	TEQAAVAR	P00748	3328,40
Coagulation factor XII	VVGGLVALR	P00748	1774,87
Complement C1q subcomponent subunit A	PAFSAIR	P02745	65,82
Complement C1q subcomponent subunit B	IAFSATR	P02746	1374,02
Complement C1q subcomponent subunit C	FQSVFTVTR	P02747	1609,25
Complement C1r subcomponent	GLTLHLK	P00736	3024,37

Complement C1r subcomponent	YTTEIIK	P00736	1197,82
Complement C1s subcomponent	TNFDNDIALVR	P09871	2356,84
Complement C2	HAFILQDTK	P06681	791,73
Complement C2	TAVDHIR	P06681	1043,17
Complement C3	SGIPIVTSPYQIHFTK	P01024	95286,28
Complement C3	TGLQEVEVK	P01024	18837,72
Complement C4-B	GSFEFPVGDAVSK	P0C0L5	10706,43
Complement C4-B	VGDTLNLNLR	P0C0L5	13478,83
Complement C5	VFQFLEK	P01031	1561,24
Complement component C8 alpha chain	HTSLGPLEAK	P07357	2246,28
Complement component C8 beta chain	SDLEVAHYK	P07358	1001,27
Complement component C9	LSPIYNLVPVK	P02748	2939,00
Complement component C9	VVEESELAR	P02748	2607,78
Complement factor B	EELLPAQDIK	P00751	1609,05
Complement factor D	THHDGAITER	P00746	612,65
Complement factor I	HGNTDSEGIVEVK	P05156	1890,56
Corticosteroid-binding globulin	ITQDAQLK	P08185	4063,99
C-reactive protein	AFVFPK	P02741	85,43
Cystatin-C	ALDFAVGEYNK	P01034	98,77
Fibrinogen alpha chain	ESSSHHPGIAEFPSR	P02671	52379,97
Fibrinogen alpha chain	GSESGIFTNTK	P02671	85669,86
Fibronectin	HTSVQTTSSGSGPFTDVR	P02751	13157,67
Fibronectin	SSPVVIDASTAIDAPSNLR	P02751	15084,41
Fibulin-1	GYHLNEEGTR	P23142	1619,46
Fibulin-1	TGYYFDGISR	P23142	1408,37
Gelsolin	AGALNSNDAFVLK	P06396	4068,25
Gelsolin	TASDFITK	P06396	4944,05
Haptoglobin	DYAEVGR	P00738	3277,19
Haptoglobin	HYEGSTVPEK	P00738	16686,91
Hemoglobin subunit alpha	TYFPHFDLSHGSAQVK	P69905	1474,04
Hemoglobin subunit alpha	VGAHAGEYGAEALER	P69905	1734,60

Hemopexin	NFPSPVDAAFR	P02790	23791,44
Heparin cofactor 2	SVNDLYIQK	P05546	1470,02
Heparin cofactor 2	TLEAQLTPR	P05546	8538,78
Insulin-like growth factor-binding protein 3	FLNVLSPR	P17936	195,86
Insulin-like growth factor-binding protein complex acid labile subunit	LAELPADALGPLQR	P35858	957,75
Inter-alpha-trypsin inhibitor heavy chain H1	GSLVQASEANLQAAQDFVR	P19827	3395,57
Inter-alpha-trypsin inhibitor heavy chain H2	FYNQVSTPLLR	P19823	14073,25
Inter-alpha-trypsin inhibitor heavy chain H2	SLAPTAAAK	P19823	7967,72
Lipopolysaccharide-binding protein	ITLPDFTGDLR	P18428	93,81
Lumican	FNALQYLR	P51884	296,66
Neuropilin-2	ALQVVR	O60462	1122,32
Pigment epithelium-derived factor	TVQAVLTVPK	P36955	31071,46
Protein AMBP	ETLLQDFR	P02760	3091,12
Protein AMBP	HHGPTITAK	P02760	2508,66
Prothrombin	ELLESYIDGR	P00734	7205,79
Prothrombin	TATSEYQTFFNPR	P00734	10760,68
Retinol-binding protein 4	YWGVASFLQK	P02753	590,56
Serotransferrin	DGAGDVAFVK	P02787	48334,64
Serotransferrin	EGYYGYTGAFR	P02787	52924,68
Serum amyloid A-4 protein	GNYDAAQR	P35542	1351,12
Sex hormone-binding globulin	IALGGLLFPASNLR	P04278	201,10
Sex hormone-binding globulin	TSSSFEVR	P04278	1578,44
Thyroxine-binding globulin	AVLHIGEK	P05543	1099,08
Transthyretin	ALGISPFHEHAEVVFTANDSGPR	P02766	24084,80
Vasorin	ESHVTLASPEETR	Q6EMK4	1166,84
Vasorin	YLQGSSVQLR	Q6EMK4	795,73
Vitamin D-binding protein	ELPEHTVK	P02774	38192,37
Vitamin D-binding protein	THLPEVFLSK	P02774	14228,53
Vitamin K-dependent protein C	LGEYDLR	P04070	416,68

Vitamin K-dependent protein S	VYFAGFPR	P07225	2531,04

Some additional targets were added on this panel. One more peptide was added to C-reactive protein (ESDTSYVSL[$^{13}C_6$]K), and one transthyretin peptide (AADDTWEPFASGK[$^{13}C_6$, $^{15}N_2$]), one albumin peptide (L[$^{13}C_6$]VNEVTEFAK), and two orosomucoid (ORM) or Alpha-1-acid glycoprotein 1 (AGA) peptides (TEDTIFL[$^{13}C_6$]R; WFYIASAFR[$^{13}C_6$, $^{15}N_4$]) were also added. SIS peptides were produced by PeptideSynthetics (Fareham, UK) with a purity up to 98%. Home made mix was spiked into the digested samples with 5 fmol of ESDTSYVSL[$^{13}C_6$]K; 10 fmol of AADDTWEPFASGK[$^{13}C_6$, $^{15}N_2$]; 50 fmol of L[$^{13}C_6$]VNEVTEFAK; 10 fmol of TEDTIFL[$^{13}C_6$]R; and 200 fmol of WFYIASAFR[$^{13}C_6$, $^{15}N_4$]).

Limit of quantification on MS assay

Performances were provided with the PeptiQuant™ Biomarker Assessment Kit (BAK-76) from CIL (Sup Table3)

To the added peptides, an increasing quantity of heavy peptides were added in digested normal plasma to determine the LOQ. LOQ was determined by the lowest points with a deviation from target of less than 20%. These analyses were performed in duplicates. L[$^{13}C_6$]VNEVTEFAK was spiked at 0, 4, 10, 20, 30 and 37.5 fmol in 2µL of digested plasma. AADDTWEPFASGK[$^{13}C_6$, $^{15}N_2$] was spiked at 0, 0.2, 0.4, 0.6, and 0.8 fmol in 2µL of digested plasma. ESDTSYVSL[$^{13}C_6$]K was spiked at 0, 0.01, 0.2, 0.6, and 2.4 fmol in 2µL of digested plasma. TEDTIFL[$^{13}C_6$]R was spiked at 0, 0.8, 1.6, 3.2, and 4.8 fmol in 2µL of digested plasma. WFYIASAFR[$^{13}C_6$, $^{15}N_4$] was spiked at 0, 16, 24, 32, 80 and 120 fmol in 2µL of digested plasma.

Precision study

Intra-assay variability was calculated on replicate analysis of plasma and DBS samples.

Inter-assay variability was calculated on 8 QC of Normal reference plasma. A coefficient of variation was calculated on these 8 values.
